# Gastroesophageal Reflux in Relation to Adenocarcinomas of the Esophagus: A Pooled Analysis from the Barrett’s and Esophageal Adenocarcinoma Consortium (BEACON)

**DOI:** 10.1371/journal.pone.0103508

**Published:** 2014-07-30

**Authors:** Michael B. Cook, Douglas A. Corley, Liam J. Murray, Linda M. Liao, Farin Kamangar, Weimin Ye, Marilie D. Gammon, Harvey A. Risch, Alan G. Casson, Neal D. Freedman, Wong-Ho Chow, Anna H. Wu, Leslie Bernstein, Olof Nyrén, Nirmala Pandeya, David C. Whiteman, Thomas L. Vaughan

**Affiliations:** 1 Division of Cancer Epidemiology and Genetics, National Cancer Institute, NIH, DHHS, Bethesda, Maryland, United States of America; 2 Division of Research and Oakland Medical Center, Kaiser Permanente, Northern California, Oakland, California, United States of America; 3 Centre for Public Health, Queen’s University, Belfast, Northern Ireland; 4 Department of Public Health Analysis, School of Community Health and Policy, Morgan State University, Baltimore, Maryland, United States of America; 5 Department of Medical Epidemiology and Biostatistics, Karolinska Institutet, Stockholm, Sweden; 6 Department of Epidemiology, University of North Carolina School of Public Health, Chapel Hill, North Carolina, United States of America; 7 Yale University School of Medicine, Department of Epidemiology and Public Health, New Haven, Connecticut, United States of America; 8 Department of Surgery, University of Saskatchewan, Saskatoon, Saskatchewan, Canada; 9 The University of Texas MD Anderson Cancer Center, Department of Epidemiology, Houston, Texas, United States of America; 10 Department of Preventive Medicine, Keck School of Medicine, University of Southern California/Norris Comprehensive Cancer Center, Los Angeles, California, United States of America; 11 Department of Population Sciences, Beckman Research Institute and City of Hope Comprehensive Cancer Center, Duarte, California, United States of America; 12 QIMR Berghofer Medical Research Institute, Brisbane, Australia; 13 Program in Epidemiology, Fred Hutchinson Cancer Research Center, Seattle, Washington, United States of America; Sapporo Medical University, Japan

## Abstract

**Background:**

Previous studies have evidenced an association between gastroesophageal reflux and esophageal adenocarcinoma (EA). It is unknown to what extent these associations vary by population, age, sex, body mass index, and cigarette smoking, or whether duration and frequency of symptoms interact in predicting risk. The Barrett’s and Esophageal Adenocarcinoma Consortium (BEACON) allowed an in-depth assessment of these issues.

**Methods:**

Detailed information on heartburn and regurgitation symptoms and covariates were available from five BEACON case-control studies of EA and esophagogastric junction adenocarcinoma (EGJA). We conducted single-study multivariable logistic regressions followed by random-effects meta-analysis. Stratified analyses, meta-regressions, and sensitivity analyses were also conducted.

**Results:**

Five studies provided 1,128 EA cases, 1,229 EGJA cases, and 4,057 controls for analysis. All summary estimates indicated positive, significant associations between heartburn/regurgitation symptoms and EA. Increasing heartburn duration was associated with increasing EA risk; odds ratios were 2.80, 3.85, and 6.24 for symptom durations of <10 years, 10 to <20 years, and ≥20 years. Associations with EGJA were slighter weaker, but still statistically significant for those with the highest exposure. Both frequency and duration of heartburn/regurgitation symptoms were independently associated with higher risk. We observed similar strengths of associations when stratified by age, sex, cigarette smoking, and body mass index.

**Conclusions:**

This analysis indicates that the association between heartburn/regurgitation symptoms and EA is strong, increases with increased duration and/or frequency, and is consistent across major risk factors. Weaker associations for EGJA suggest that this cancer site has a dissimilar pathogenesis or represents a mixed population of patients.

## Introduction

The association between gastroesophageal reflux and inflammation of the distal esophageal mucosa was first expounded by Winkelstein in 1935 [1]. Barrett himself acknowledged that gastroesophageal reflux may be a cause of the eponymously titled metaplastic lesion that precedes adenocarcinoma [2], and future human observations [3] and animal experiments [4] were to provide evidence for such. Concurrent with these developments was the proposition, derived from clinical observation, that gastroesophageal reflux may predispose to cancer of the distal esophagus [5]. Three studies, completed in the 1990s, provided strong and seminal epidemiologic evidence for this hypothesis [6]–[8], and subsequent studies provided confirmatory evidence for the association between gastroesophageal reflux and adenocarcinomas of the esophagus [9]–[12]. However, it is unknown to what extent these associations vary by population using harmonized adjusted models. Furthermore, investigations of whether these associations differ with respect to age, sex, body mass index (BMI), cigarette smoking, and anti-reflux medications have been limited due to small numbers upon stratification. Lastly, the interplay between duration and frequency of exposure with respect to risk of esophageal adenocarcinomas is unclear. Therefore, we assessed whether heartburn and regurgitation exposures were associated with esophageal adenocarcinoma (EA) and esophagogastric junction adenocarcinoma (EGJA) by pooling, harmonizing, and analyzing detailed individual participant data from five case-control studies in the international Barrett’s and Esophageal Adenocarcinoma Consortium (BEACON, http://beacon.tlvnet.net/).

## Methodology

### Study Population

The BEACON consortium was formed in 2005 with support from the U.S. National Cancer Institute. It is composed of investigators from around the world and brings together population-based case-control and cohort studies of Barrett’s esophagus, EA and EGJA. The primary objectives of BEACON are to facilitate well-powered, combined investigations of risk factors in relation to these diseases, as well as aid in the development of new studies of etiology, prevention and survival.

Twelve BEACON studies included in a pooled analysis of tobacco smoking in relation to adenocarcinomas of the esophagus have been described previously [13]. Five of these studies were able to provide information on heartburn and regurgitation exposures: the nationwide *Australian Cancer Study* (Esophageal Cancer Component) [11]; *FINBAR* (Factors INfluencing the Barrett’s/Adenocarcinoma Relationship) study, based in Ireland [12]; Los Angeles County Multi-ethnic Case–control Study [14]; a nationwide Swedish study of esophageal cancer and esophagogastric junction adenocarcinoma [15]; and the United States (US) Multi-center Study [16] (See File S1 for further details).

In combination, these five studies provided 1,197 EA cases, 1,317 EGJA cases, and 4,711 population-based controls. We restricted the analytic population to white non-Hispanics, due to the relatively small number of non-White, non-Hispanic case patients (17 Black, 101 Hispanic, 39 other race or ethnic groups). After these exclusions there remained 1,128 EA cases, 1,229 EGJA cases, and 4,057 controls for analysis. Data acquisition and data pooling for each study were approved by the Institutional Review Board or Research Ethics Committee of the institute(s) sponsoring each study.

### Study Variables

Self-reported questionnaires were administered at or near the time of cancer diagnosis for case patients and at time of recruitment for control subjects. The two primary exposures for the study were symptoms of heartburn and regurgitation. Heartburn symptoms related to burning or aching pain behind the breastbone/sternum not due to heart problems, and regurgitation symptoms were commonly specified as a sour taste resulting from regurgitation of acid, bile or other stomach contents into the mouth. The questions used by each study to ascertain these exposures are shown in Table 1 in File S1. Heartburn and regurgitation symptoms were harmonized as recurrent/not recurrent (dichotomous using a frequency of weekly or greater for ‘recurrent’), categories of duration of exposure (0, 1–9, 10–19, 20+ years) and categories of frequency of exposure (never, <monthly, monthly to <weekly, weekly to <daily, ≥daily). The term GERD (gastroesophageal reflux disease) will be used to refer to the combined exposure of heartburn or regurgitation. This combined exposure was assessed given that heartburn and regurgitation symptoms essentially reflect a similar exposure; namely the exposure of the esophagus to gastric juice.

Other variables included in our analyses were age, sex, education, BMI (weight divided by square of height [kg/m^2^]), cigarette smoking, alcohol consumption, use of proton pump inhibitors, H_2_ receptor antagonists, antacids, any anti-reflux medications (catch-all variable), and non-steroidal anti-inflammatory drugs, as well as study-specific variables (study center for US Multi-center Study and country of birth for Los Angeles County Multi-ethnic Case–control Study).

### Statistical Analysis

We used a two-step analytic approach. First, we used multivariable logistic regression models to estimate study-specific odds ratios (ORs) and 95% confidence intervals (CIs) of the association between exposure and outcome in each study. Second, the study-specific ORs were pooled using random-effects meta-analysis to generate summary ORs [17]. We excluded study-specific results from a particular meta-analysis if the underlying model from that study failed to converge.

Study-specific, minimally adjusted logistic regression models included the covariates age (categorical: <50, 50–59, 60–69, ≥70 years), sex, and study-specific variables (where appropriate). In each study, we assessed whether any of the following variables changed pooled or study-specific dichotomous exposure estimates (ORs) by >10%: BMI, height, recent weight, cigarette smoking, alcohol consumption, non-steroidal anti-inflammatory drugs, education, fruit consumption, and vegetable consumption. Only pack-years of cigarette smoking altered estimates of a single study (FINBAR) by >10%. However, in addition to those variables included in the minimally adjusted models, we included the following covariates in all study-specific maximally adjusted models given previous evidence of associations between these exposures and adenocarcinomas of the esophagus: BMI (categorical: <25, 25–29.9, ≥30) [18], education (study-specific) [19], [20], alcohol consumption (categorical: <7, 7–20, ≥21 drinks per week) [21], and cigarette smoking (categorical: 0, 1–14, 15–29, 30–44, ≥45 pack-years) [13]. Results were not materially different between minimally and maximally adjusted models, thus we present only the latter results.

To investigate potential effect-modification (and between-study heterogeneity) we conducted analyses of recurrent heartburn and/or recurrent regurgitation stratified analyses by age (<60, 60–69, ≥70 years), sex (male/female), cigarette smoking (ever/never), and BMI (<25, 25–29, ≥30) as these are known risk factors for esophageal adenocarcinomas. The statistical significance of potential effect-modifiers was assessed by a two-step analysis of product-terms using dichotomous (cigarette smoking, sex) or continuous (age, BMI) variables combined with the primary exposures of interest. To further investigate between-study heterogeneity, we also conducted meta-regressions of anti-gastroesophageal reflux medications (e.g., proton pump inhibitors, H_2_ receptor antagonists, antacids, and any anti-reflux medications) and mid-year of recruitment using the STATA metareg command with 5,000 Monte Carlo permutations to generate each p value [22]. A false-discovery rate method was used to control the type I error [23]. Lastly, we also conducted sensitivity analyses whereby each study was omitted in-turn with re-estimation of the association to determine if any single study dominated a summary OR. The *I^2^* value and its 95% uncertainty interval were used to estimate the percentage of total variation across studies due to heterogeneity [24]. An *I^2^* statistic of 0% indicates no observed heterogeneity, whereas larger values indicate increasing heterogeneity. All analyses were performed using STATA software, version 12 (StataCorp LP, College Station, TX). All statistical tests were two-sided. *P* values less than 0.05 were considered to be statistically significant.

## Results

### Descriptive statistics of Study Populations

There were 1,128 EA cases, 1,229 EGJA cases, and 4,057 controls available for analysis (Table 1). The cases and controls were predominantly male (66–87%), with a median age of approximately 65 years old. Cases were more likely than controls to smoke cigarettes and, of those who did, total exposure was also greater, using the exposure metric of pack-years of cigarette smoking. The proportions that reported recurrent (weekly or greater) heartburn and/or recurrent regurgitation were greatest in the EA group, then the EGJA group, and lowest amongst the controls; anti-gastroesophageal reflux medications displayed a similar pattern.

**Table 1:** Descriptors of Participants Eligible for Analysis of Heartburn/Regurgitation in Relation to Esophageal Adenocarcinoma and Esophagogastric Junction Adenocarcinoma.

Variable	All StudiesCombined			AustralianCancer Study			USMulti-Center Study			FINBARStudy			Los AngelesMulti-Ethnic Study			SwedishEsophagealCancer Study		
	Control(n = 4,057)	EA(n = 1,128)	EGJA(n = 1,229)	Control(N = 1,512)	EA(N = 359)	EGJA(N = 419)	Control(N = 624)	EA(N = 278)	EGJA(N = 246)	Control(N = 260)	EA(N = 131)	EGJA(N = 92)	Control(N = 841)	EA(N = 171)	EGJA(N = 210)	Control(N = 820)	EA(N = 189)	EGJA(N = 262)
Age (years)	64 (55, 71)	65 (57, 72)	65 (56, 71)	62 (53, 69)	64 (57, 71)	64 (57, 71)	64 (57, 71)	66 (56, 73)	66 (57, 72)	65 (53, 72)	66 (57, 73)	65 (56, 71)	63 (54, 69)	62 (57, 69)	65 (56, 69)	68 (59, 74)	69 (62, 73)	66 (57, 73)
Male (%)	75 (74, 77)	87 (85, 89)	86 (84, 87)	66 (64, 69)	91 (87, 94)	87 (83, 90)	80 (77, 83)	83 (79, 88)	85 (80, 89)	85 (80, 89)	84 (78, 90)	87 (80, 94)	78 (76, 81)	89 (85, 94)	84 (79, 89)	83 (80, 85)	87 (83, 92)	85 (81, 89)
Body mass index (kg/m2)	25 (23, 28)	27 (24, 30)	26 (24, 30)	26 (24, 29)	28 (26, 32)	28 (25, 31)	25 (23, 27)	25 (24, 28)	25 (23, 28)	27 (24, 29)	28 (25, 31)	28 (26, 31)	25 (23, 28)	26 (23, 29)	26 (23, 30)	24 (22, 25)	25 (24, 28)	25 (23, 27)
Cigarette smoker ever (%)	59 (57, 60)	75 (72, 78)	78 (76, 80)	55 (52, 57)	75 (70, 79)	77 (73, 81)	69 (65, 73)	79 (74, 84)	81 (76, 86)	60 (54, 66)	80 (73, 87)	79 (70, 87)	63 (60, 66)	80 (73, 86)	75 (69, 81)	55 (52, 59)	63 (56, 70)	78 (73, 83)
Cigarettes (pack-years)^1^	24 (10, 43)	34 (17, 54)	32 (18, 51)	20 (8, 40)	31 (15, 52)	30 (16, 49)	33 (14, 54)	41 (21, 68)	38 (25, 60)	29 (12, 50)	40 (20, 65)	37 (26, 60)	31 (13, 51)	36 (21, 58)	43 (22, 74)	16 (6, 32)	19 (8, 32)	24 (10, 38)
Alcohol ever (%)	88 (87, 89)	87 (85, 89)	89 (87, 91)	89 (87, 91)	93 (90, 96)	92 (90, 95)	94 (92, 96)	92 (89, 96)	95 (92, 98)	71 (66, 77)	68 (59, 76)	60 (49, 70)	83 (81, 86)	84 (79, 90)	84 (79, 89)	92 (90, 93)	85 (79, 90)	92 (89, 95)
Alcohol (drinks per week)^1^	9 (3, 19)	11 (4, 26)	10 (4, 23)	9 (4, 19)	13 (6, 25)	12 (5, 24)	9 (3, 21)	13 (4, 28)	10 (5, 24)	21 (5, 47)	13 (6, 36)	12 (4, 27)	9 (3, 18)	9 (2, 21)	8 (3, 28)			
Education (% ≥high-school)	65 (64, 67)	60 (57, 62)	62 (59, 65)	59 (56, 61)	54 (48, 59)	59 (55, 64)	83 (80, 86)	78 (74, 83)	80 (75, 85)	58 (52, 64)	46 (37, 54)	39 (29, 49)	93 (91, 94)	90 (86, 95)	88 (84, 93)	39 (36, 42)	25 (19, 31)	36 (30, 41)
Recurrent Heartburn (%)	12 (11, 13)	40 (37, 43)	26 (24, 29)	11 (10, 13)	38 (33, 43)	28 (24, 33)	13 (10, 16)	33 (27, 39)	20 (15, 25)	17 (13, 22)	48 (40, 57)	40 (30, 50)	13 (11, 15)	41 (33, 48)	32 (26, 39)	10 (8, 12)	47 (40, 55)	20 (15, 25)
Recurrent Regurgitation (%)	10 (9, 11)	34 (31, 37)	21 (19, 24)	8 (7, 10)	33 (28, 38)	25 (21, 30)	12 (9, 14)	30 (24, 35)	16 (11, 20)	11 (7, 15)	35 (26, 43)	24 (15, 33)	15 (13, 18)	47 (40, 55)	30 (24, 37)	6 (4, 7)	30 (23, 37)	11 (8, 15)
Recurrent Heartburn or Regurgitation (%)	17 (15, 18)	50 (47, 53)	35 (32, 37)	15 (13, 17)	47 (42, 52)	39 (35, 44)	20 (17, 24)	46 (40, 52)	26 (21, 32)	18 (14, 23)	51 (42, 59)	45 (34, 55)	20 (17, 23)	58 (51, 66)	44 (37, 51)	13 (10, 15)	54 (47, 61)	23 (18, 28)
PPI use ever (%)	8 (7, 9)	20 (17, 23)	20 (17, 22)	15 (13, 17)	40 (35, 45)	39 (34, 44)	1 (0, 2)	3 (1, 5)	2 (0, 4)	11 (7, 15)	15 (9, 22)	14 (7, 22)	1 (0, 1)	7 (3, 11)	3 (0, 5)			
H2RA use ever (%)	17 (16, 18)	29 (26, 32)	25 (23, 28)	18 (16, 20)	36 (31, 41)	31 (27, 36)	17 (14, 20)	23 (18, 28)	20 (15, 25)	11 (7, 15)	20 (13, 27)	14 (7, 21)	17 (15, 20)	32 (24, 39)	24 (18, 31)			
Antacid use ever (%)	48 (46, 50)	59 (56, 62)	58 (55, 62)	43 (40, 45)	70 (65, 75)	69 (65, 74)	24 (21, 28)	50 (44, 56)	39 (32, 45)	7 (4, 10)	7 (2, 11)	2 (−1, 5)	91 (90, 93)	94 (91, 98)	88 (83, 93)			
Any antireflux medication use ever* (%)	50 (48, 51)	73 (70, 75)	62 (59, 65)	51 (48, 54)	80 (76, 84)	76 (72, 80)	38 (34, 42)	63 (57, 68)	49 (43, 55)	30 (25, 36)	59 (50, 67)	57 (46, 67)	92 (90, 94)	95 (92, 99)	88 (83, 92)	22 (19, 25)	65 (58, 72)	36 (30, 42)

The median and interquartile range of each variable are provided, unless the variable is stated to be a percentage in which case the percentage and 95 percent confidence interval are provided. *This includes PPIs, H2RAs, antacids, and non-specific questions about anti-reflux medications/remedies. Abbreviations: EA, esophageal adenocarcinoma; EGJA, esophagogastric junction adenocarcinoma; FINBAR, Factors Influencing the Barrett’s Adenocarcinoma Relationship (FINBAR) Study. 1Among those exposed.

### Heartburn or Regurgitation Exposures

Table 2 shows the relationship between the presence of recurrent heartburn and/or recurrent regurgitation and risk of adenocarcinomas of the esophagus. Recurrent heartburn/recurrent regurgitation was associated with an approximate 5-fold statistically significant increased risk of EA. For EGJA, the associations were also statistically significant albeit slightly weaker than those for EA at around 2-fold increased risk. Of note was the moderate-to-high heterogeneity (*I^2^*) associated with each summary risk estimate, with *I^2^s* ranging from 47% to 84%.

**Table 2:** Associations between recurrent heartburn and/or recurrent regurgitation and adenocarcinomas of the esophagus.

Analysis	EA				EGJA			
	Case(n)	Control(n)	OR (95%CI)	*I^2^*(95%UI)	Case(n)	Control(n)	OR (95%CI)	*I^2^*(95%UI)
**Heartburn**								
Not-recurrent	606	3,215	*Referent*		807	3,215	*Referent*	
Recurrent	391	419	4.64 (3.28−6.57)	74 (34−89)	288	419	2.40 (1.86−3.10)	47 (0−81)
**Regurgitation**								
Not-recurrent	613	3,256	*Referent*		834	3,256	*Referent*	
Recurrent	379	409	4.57 (3.43−6.08)	55 (0−83)	254	409	1.86 (1.14−3.06)	82 (59−92)
**Heartburn or Regurgitation**								
Not-recurrent	489	2,971	*Referent*		706	2,971	*Referent*	
Recurrent	486	577	4.81 (3.39−6.82)	76 (42−90)	371	577	2.26 (1.46−3.48)	84 (65−93)

Adjusted for age, sex, BMI, education, alcohol consumption, cigarette smoking, and study-specific variables. Abbreviations: AA, all adenocarcinoma; CI, confidence interval; EA, esophageal adenocarcinoma; EGJA, esophagogastric junction adenocarcinoma; OR.

**Figure 1:**
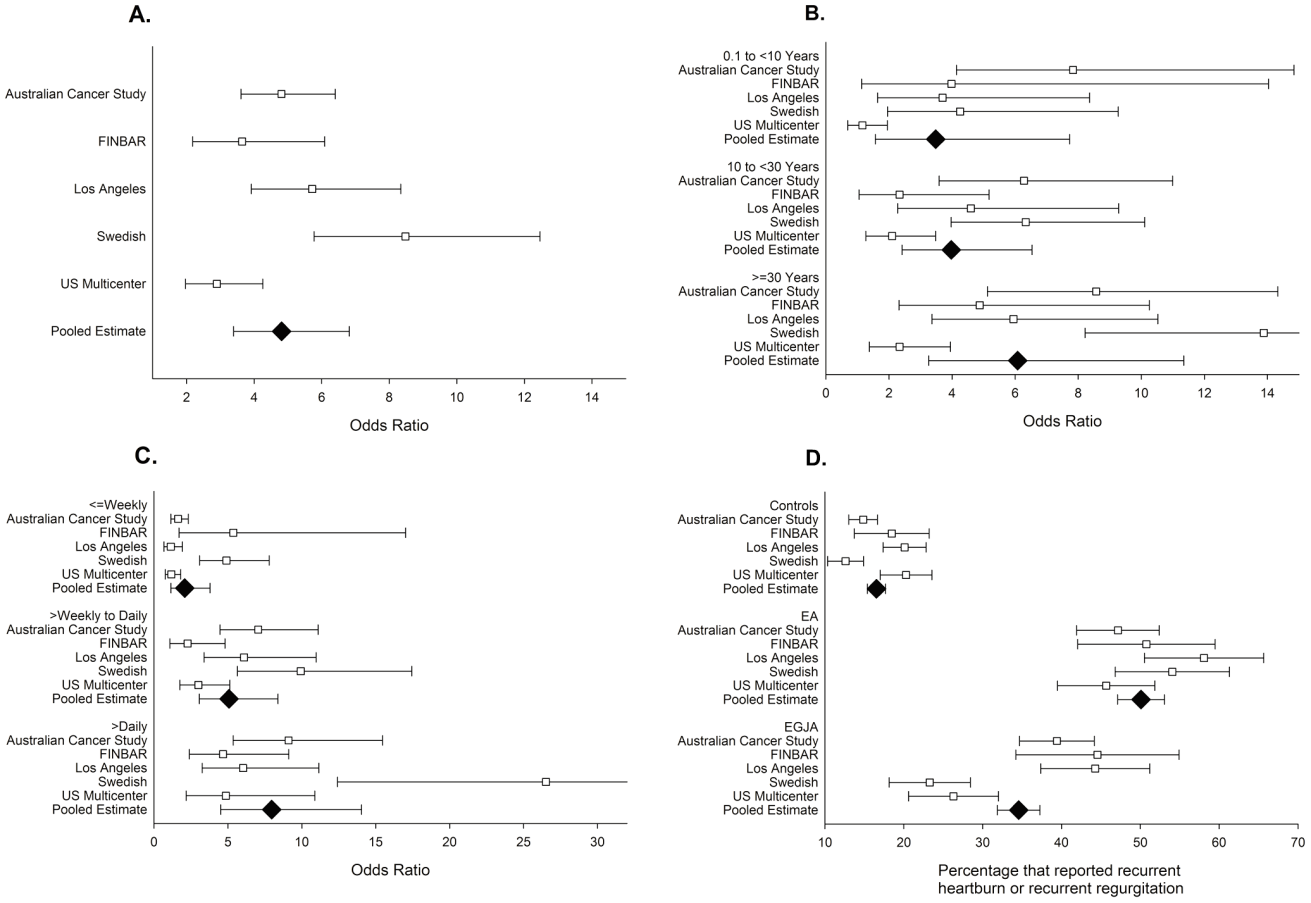
Forest plots of associations between heartburn and regurgitation exposures in relation to case and control groups in BEACON. A: The association between recurrent heartburn or recurrent regurgitation in relation to esophageal adenocarcinoma. B: The association between heartburn and regurgitation duration in relation to esophageal adenocarcinoma. C: The association between heartburn and regurgitation frequency in relation to esophageal adenocarcinoma. D. The frequency of recurrent heartburn or recurrent regurgitation exposure in case and control groups by study. For each plot each white square represents the study-specific odds ratio (A–C) or prevalence of exposure (D) and the black diamond represents the overall estimate. The arms of each symbol portray the 95% confidence intervals.

Associations between increasing duration and frequency of gastroesophageal reflux in relation to adenocarcinomas of the esophagus are shown in Tables 3 and 4, respectively, as well as in Figure 1. Note that these analyses are not restricted to those with recurrent (weekly) heartburn/regurgitation. In Tables 3 and 4 it is evident that as heartburn or regurgitation exposure increases so does the strength of the association with EA. For example, ORs for increasing heartburn duration were 2.80 (95%CI: 1.60, 4.91), 3.85 (95%CI: 2.93, 5.07), and 6.24 (95%CI: 3.37, 11.55) for durations of exposure of <10 years, 10 to <20 years, and ≥20 years, respectively, all compared with those not experiencing symptoms. The associations of increasing gastroesophageal reflux exposures with EGJA were, relative to those for EA, much weaker, but still statistically significant in a majority of the highest exposure categories. Heterogeneity was often moderate (∼50%) to high (∼75%), but with wide uncertainty intervals. In joint-effects models of increasing duration and increasing frequency of gastroesophageal reflux symptoms, it was clear that both factors play a role in risk of adenocarcinomas of the esophagus with some indication that frequency may be slightly more important, given the categorical cut-points assessed (Table 5 and Tables 2–5 in File S1).

**Table 3:** Associations between heartburn and/or regurgitation duration and adenocarcinomas of the esophagus.

Analysis	EA				EGJA			
	Case(n)	Control(n)	OR (95%CI)	*I^2^*(95%UI)	Case(n)	Control(n)	OR (95%CI)	*I^2^*(95%UI)
**Heartburn Duration (years)**								
Never	410	2,339	*Referent*		612	2,339	*Referent*	
0.1 to <10	87	174	2.80 (1.60−4.91)	68 (16−88)	93	174	1.97 (1.30−2.98)	44 (0−79)
10 to <20	124	198	3.85 (2.93−5.07)	0 (0−31)	70	198	1.30 (0.63−2.68)	78 (47−91)
≥20	242	230	6.24 (3.37−11.55)	85 (66−93)	187	230	2.85 (1.61−5.05)	81 (56−92)
**Regurgitation Duration (years)**								
Never	393	2,215	*Referent*		594	2,215	*Referent*	
0.1 to <10	126	227	2.69 (1.49−4.83)	75 (40−90)	117	227	1.70 (0.65−4.45)	91 (83−96)
10 to <20	114	185	4.18 (2.37−7.37)	71 (26−89)	70	185	1.44 (0.68−3.08)	79 (49−91)
≥20	222	257	4.39 (2.34−8.25)	84 (63−93)	151	257	1.64 (0.81−3.31)	84 (64−93)
**Heartburn & Regurgitation Duration (years)**								
Never	290	1,820	*Referent*		467	1,820	*Referent*	
0.1 to <10	87	181	3.48 (1.56−7.73)	82 (60−92)	95	181	2.38 (0.77−7.35)	92 (84−96)
10 to <30	146	226	3.97 (2.41−6.54)	72 (30−89)	118	226	1.92 (0.99−3.72)	84 (65−93)
≥30	258	303	6.08 (3.26−11.34)	83 (63−93)	170	303	2.23 (1.22−4.08)	81 (55−92)

Adjusted for age, sex, BMI, education, alcohol consumption, cigarette smoking, and study-specific variables. Abbreviations: AA, all adenocarcinoma; CI, confidence interval; EA, esophageal adenocarcinoma; EGJA, esophagogastric junction adenocarcinoma; OR.

**Table 4:** Associations between heartburn and/or regurgitation frequency and adenocarcinomas of the esophagus.

Analysis	EA				EGJA			
	Case(n)	Control(n)	OR (95%CI)	*I^2^*(95%UI)	Case(n)	Control(n)	OR (95%CI)	*I^2^*(95%UI)
**Heartburn Frequency**								
Never	410	2,339	*Referent*		612	2,339	*Referent*	
<monthly	83	624	0.91 (0.68−1.21)	0 (0−59)	86	624	0.58 (0.43−0.78)	8 (0−86)
Monthly to <weekly	113	252	2.90 (1.78−4.72)	56 (0−85)	109	252	1.76 (1.24−2.50)	26 (0−71)
Weekly to <daily	202	281	4.20 (2.76−6.40)	68 (18−88)	173	281	2.19 (1.45−3.29)	66 (11−87)
≥Daily	189	138	7.42 (4.23−13.02)	76 (41−90)	115	138	2.84 (2.10−3.85)	6 (0−81)
**Regurgitation Frequency**								
Never	393	2,215	*Referent*		594	2,215	*Referent*	
<monthly	91	768	0.71 (0.48−1.04)	37 (0−78)	114	768	0.57 (0.26−1.24)	87 (68−94)
Monthly to <weekly	129	270	3.13 (2.14−4.58)	40 (0−78)	126	267	1.90 (1.47−2.47)	0 (0−83)
Weekly to <daily	233	276	5.07 (3.51−7.32)	55 (0−83)	151	276	1.60 (0.73−3.52)	88 (75−94)
≥Daily	146	133	4.94 (3.37−7.24)	35 (0−75)	103	133	2.29 (1.43−3.66)	51 (0−82)
**Heartburn & Regurgitation Frequency**								
Never	290	1,820	*Referent*		467	1,820	*Referent*	
≤Weekly	262	1,269	2.08 (1.14−3.79)	86 (69−94)	305	1,269	1.26 (0.70−2.27)	88 (74−94)
>Weekly to Daily	230	304	5.07 (3.07−8.38)	75 (37−90)	187	304	2.14 (1.08−4.23)	86 (68−93)
>Daily	186	148	7.96 (4.51−14.04)	73 (31−89)	106	148	2.64 (1.66−4.18)	51 (0−82)

Adjusted for age, sex, BMI, education, alcohol consumption, cigarette smoking, and study-specific variables. Abbreviations: AA, all adenocarcinoma; CI, confidence interval; EA, esophageal adenocarcinoma; EGJA, esophagogastric junction adenocarcinoma; OR.

**Table 5:** Associations between heartburn & regurgitation frequency, duration and esophageal adenocarcinoma and esophagogastric junction adenocarcinoma.

		Heartburn & Regurgitation Frequency		
		Never	<weekly	≥weekly
**EA**				
**Heartburn** **& Regurgitation** **Duration (years)**	**Never**	*Referent*	*-*	*-*
	**0.1 to <20**	-	3.13 (95%CI: 1.49–6.56; I2 = 84)	4.75 (95%CI: 2.66–8.47; I2 = 72)
	**≥20**	-	1.51 (95%CI: 0.77–2.96; I2 = 35)	9.27 (95%CI: 5.02–17.10; I2 = 78)
**EGJA**				
**Heartburn** **& Regurgitation** **Duration (years)**	**Never**	*Referent*	*-*	*-*
	**0.1 to <20**	-	1.92 (95%CI: 0.80–4.59; I2 = 90)	2.20 (95%CI: 1.11–4.37; I2 = 79)
	**≥20**	-	1.55 (95%CI: 0.73–3.25; I2 = 59)	2.55 (95%CI: 1.32–4.92; I2 = 76)

Adjusted for age, sex, BMI, education, alcohol consumption, cigarette smoking, and study-specific variables. Abbreviations: CI, confidence interval.

### Sensitivity Analyses

Sensitivity analyses demonstrated that the Swedish study was a major contributor to the heterogeneity in analyses of heartburn in relation to EA; excluding this study attenuated associations slightly and also lowered the heterogeneity (OR*_recurrent heartburn_* = 4.04, 95%CI: 3.13, 5.22, *I^2^* = 39%; OR*_duration category 2_* = 2.45, 95%CI: 1.31, 4.60, *I^2^* = 71%; OR*_duration category 3_* = 3.78, 95%CI: 2.75, 5.19, *I^2^* = 0%; OR*_duration category 4_* = 4.75, 95%CI: 3.18, 7.09, *I^2^* = 54%; OR*_frequency category 2_* = 0.91, 95%CI: 0.68, 1.21, *I^2^* = 0%; OR*_frequency category 3_* = 2.54, 95%CI: 1.83, 3.53, *I^2^* = 10%; OR*_frequency category 4_* = 3.70, 95%CI: 2.30, 5.96, *I^2^* = 66%; OR*_frequency category 5_* = 5.70, 95%CI: 4.23, 7.67, *I^2^* = 0%). Exclusion of this study from the dichotomous recurrent/not recurrent regurgitation analysis in relation to EA also caused a reduction in estimated heterogeneity (OR*_recurrent regurgitation_* = 4.16, 95%CI: 3.18, 5.43, *I^2^* = 38%), although its exclusion had minimal impact on the moderate-to-high heterogeneity detected in the analyses of regurgitation duration and regurgitation frequency (data not shown). In analyses of recurrent heartburn and recurrent regurgitation exposures combined, the US Multi-center Study was the predominant source of the heterogeneity–although with exclusion of this study, heterogeneity for a majority of heartburn/regurgitation results remained at levels considered moderate-to-high (data not shown) and there was no effect on estimates of heartburn and regurgitation frequency. Sensitivity analyses of EGJA did not indicate any predominant source of heterogeneity.

### Effect-modification and Meta-regression Analyses

The only interaction term for effect-modification that was statistically significant at the nominal level of α = 0.05 was sex (p = 0.02) in relation to the association between recurrent heartburn and esophageal adenocarcinoma. Relationships for EA and EGJA were slightly stronger for women compared with equivalent estimates for men (Tables 6–11 in File S1). However, after adjustment for multiple testing using a false-discovery rate methodology [23], the interaction term was not deemed to be statistically significant. Although none of the other stratified analyses provided evidence for effect-modification, the analyses stratified by BMI suggested some slightly increased risks for the obese group, relative to normal and overweight groups (Tables 12–20 in File S1). Stratification by age revealed slightly stronger associations for EA in individuals aged either ≥60 to <70 years or ≥70 years, compared with individuals aged <60 years (Tables 21–29 in File S1). Stratification by cigarette smoking, suggested slightly elevated associations between recurrent heartburn and EA for never-smokers (Tables 30–35 in File S1). Meta-regressions of anti-gastroesophageal reflux medications and of mid-year of recruitment were not statistically significant after adjustment for multiple testing. These findings suggest that heterogeneity in the primary analyses was not solely due to differences in the use of anti-gastroesophageal reflux medications or to unknown period effects.

## Discussion

This analysis of BEACON data supports a strong positive association between heartburn and/or regurgitation and increased risk of adenocarcinomas of the esophagus, as well as positive dose-response relationships with increasing duration and frequency of exposure. For EA, all estimates were statistically significant and suggested that recurrent symptoms of heartburn and/or recurrent regurgitation was associated with an approximate 5-fold increased risk of EA. For EGJA the associations were weaker but still statistically significant. Increasing symptom duration was associated with greater risk of EA–risks were about 3-fold, 4-fold and 6-fold higher for symptom durations of <10 years, 10 to <20 years, and ≥20 years, respectively, all compared with no exposure (never). Associations between increased frequency/duration of heartburn/regurgitation with EGJA were weaker, but still statistically significant for the highest exposed categories. From joint effects analyses, it was apparent that both increased frequency and duration of symptoms were associated with higher risk of EA. Again, equivalent analyses for EGJA exhibited similar, albeit weaker, associations.

Although statistically significant associations for recurrent GERD (heartburn or regurgitation) exposure and cancer were observed separately for each study, there was moderate-to-high heterogeneity in the magnitude of the observed relative risk estimates, with the strongest associations often provided by the Swedish study. This was particularly evident for analyses of recurrent heartburn in relation to EA. High heterogeneity was also observed in a recent meta-analysis of gastroesophageal reflux and EA [10]. The most obvious difference of the Swedish study, which likely accounts for the more pronounced relationships between GERD and EA, is the combined consequence of relatively low recurrent exposure in controls (13%) and relatively high recurrent exposure in cases (54%) (see Figure 1). The latter is possibly explained by the fact that the Swedish study was the only study to define EGJA as adenocarcinoma with its center within 2 cm oral to, or 3 cm aboral to, the gastroesophageal junction and thus to exclude cancers “centered” in the most aboral 2 cm of the esophagus from its definition of esophageal adenocarcinoma [7]; the other included studies defined EA as any adenocarcinoma that was “centered” above the gastroesophageal junction. Furthermore, the analyses we present here of EGJA suggest these excluded tumors have a weaker association with GERD. However, the Swedish study did not provide higher estimates of GERD in relation to EGJA relative to other studies, although this grouping of tumors are known to be heterogeneous in their pathogenesis thus addition of distal EAs to the EGJA case-group may have limited effects on estimates of association. It is possible that in the Swedish study population GERD symptoms were differentially reported, relative to the other included studies, given that the questionnaire was in Swedish and the word *halsbränna* refers to a burning sensation which could occur retrosternally and/or in the upper throat. When recurrent heartburn and recurrent regurgitation variables were combined, the US Multi-center Study was the predominant contributor of heterogeneity, although even after exclusion of this study, heterogeneity remained moderate-to-high for most summary estimates. It is conceivable that GERD may vary in its carcinogenic potency in different populations for reasons such as genetic background (i.e., gene-environment interactions) and diet. For example, the composition of refluxate can affect symptom perception as well as the capacity for mucosal damage [25] and this may differ geographically.

Associations of heartburn/regurgitation in relation to EGJA were positive, but not as strong as those observed for EA. A possible reason for this is that EGJA tumors likely represent a heterogeneous groups of malignancies–some with a pathogenesis similar to that of EA and others with a pathogenesis similar to that of gastric cancer [26], [27]. As of yet, there is no method to differentiate between these two types of cancers with certainty, although suggestions based on the histology of adjacent stomach tissue may be useful in future studies of EGJA [26].

Strengths of this analysis include the availability of individual participant data which enabled harmonization of variables and statistical models, as well as permitting flexibility of analysis. This reduces the likelihood that the heterogeneity detected was a result of differences in inclusion of covariates, modeling of covariates, or choice of statistical parameters. The consortial approach enabled generation of the largest dataset yet to permit assessment of the association between gastroesophageal reflux and adenocarcinomas of the esophagus. Limitations of this analysis include the moderate-to-high heterogeneity associated with a majority of summary estimates presented–cautious interpretation as to the magnitude of these estimates is therefore warranted. It is important to note that this pooled analysis assesses self-reported symptoms of heartburn and regurgitation, yet exposure may not always elicit symptoms. However, it has been shown that symptoms are indicative of greater severity of acid reflux exposure [28]. Moreover, to differentiate between infrequent heartburn/regurgitation, which is quite common in most western populations, and symptoms which are more likely to reflect pathologic reflux, we defined recurrent exposure as being of a frequency of at least weekly. Related to this point is the fact that the presence of Barrett’s esophagus–a condition associated with gastroesophageal reflux and the recognized precursor to EA–is thought to desensitize the esophagus to such exposures. However, one would expect this to bias results towards the null, as one would expect a higher prevalence of Barrett’s esophagus in cases than population-based controls. It is conceivable that study variability in symptom exclusion period contributed to the moderate-to-high heterogeneity estimated, although the Australian Cancer Study–with the longest symptom exclusion period–was not a major source of heterogeneity. A final limitation is that case-control studies may be affected by recall bias, with esophageal cancer patients more accurately or possibly over-reporting reflux symptomatology leading to over-estimated relationships.

In conclusion, our analysis of individual participant data from the international BEACON consortium provides evidence for a strong relationship between gastroesophageal reflux exposures and adenocarcinomas of the esophagus, and indicates that longer duration and increased frequency of reflux are both associated with carcinogenic risk. Future studies should aim to ascertain gastroesophageal reflux exposures across the life-course using validated exposure assessment tools. In addition, studies are needed to further elucidate the morphological, functional, molecular and bacteriological mechanisms that link severe gastroesophageal reflux disease to cancer.

## Supporting Information

File S1 Study and exposure ascertainment information, and effect-modification and interaction analyses of gastroesophageal reflux in relation to adenocarcinomas of the esophagus.
(DOC)
