# Supplementary material for: Gastroesophageal Reflux in Relation to Adenocarcinomas of the Esophagus: A Pooled Analysis from the Barrett’s and Esophageal Adenocarcinoma Consortium (BEACON)
Source: PLoS One. 2014 Jul 30;9(7):e103508. doi: 10.1371/journal.pone.0103508 (PMC4116205; doi:10.1371/journal.pone.0103508)
Supplement: Supplementary file 1 [file pone.0103508.s001.doc]

Supplementary Table 1. Studies included in the pooled analysis and questions used to elicit heartburn and regurgitation exposures.

Study	Country	Years of Diagnosis	Symptom exclusion period prior to diagnosis (years)	Heartburn/Regurgitation/Heartburn or Regurgitation	Exposure	Response on Questionnaire in Categories or Continuous	Question (translation for non-English questionnaires)
Australian Cancer Study (esophageal cancer component), Whiteman, 2008	Australia	2001–2005	10 years for Recurrent and Frequency variables.	Heartburn	Recurrent / Non-recurrent	Categories	Have you ever had HEARTBURN? (a burning pain behind the breastbone after eating). Yes, No, Don't know. [*Recurrent defined as a few times a week, or daily from ANY of the four age categories within the valid period of the heartburn frequency question with pregnancy-only heartburn excluded*].
					Frequency	Categories	How OFTEN did you have HEARTBURN when you were: 4x6 [aged 10-19, 20-29, 30-49, 50-79] [never, less than once a month, a few times a month, a few times a week, daily, n/a] *Only the MOST CURRENT valid frequency age variable was used based on their age and the exclusion period. Pregnancy-only frequency was not used.*
					Duration	Continuous (granularity = 1/100 of a year)	Lifetime duration of FREQUENT HEARTBURN (derived)
				Regurgitation	Recurrent / Non-recurrent	Categories	Have you ever had ACID REFLUX? that is, a sour taste from acid or bile rising up into your mouth or throat. Yes, No, Don't know. [*Recurrent defined as a few times a week, or daily from ANY of the four age categories within the valid period of the heartburn frequency question with pregnancy-only heartburn excluded*].
					Frequency	Categories	How OFTEN did you have ACID REFLUX when you were: 4x6 [aged 10-19, 20-29, 30-49, 50-79] [never, less than once a month, a few times a month, a few times a week, daily, n/a] *Only the MOST CURRENT valid frequency age variable was used based on their age and the exclusion period. Pregnancy-only frequency was not used.*
					Duration	Continuous (granularity = 1/100 of a year)	Lifetime duration of FREQUENT ACID REFLUX (derived)
US Multi-Center Study, Gammon, 1997	United States	1993–1995	1 year	Heartburn	Recurrent / Non-recurrent	Categories	Before a year ago, did you ever have severe heartburn, that is, heartburn so painful that is awoke you or prevented you from sleeping? Yes, No, Don't know. [*Recurrent defined as at least once per week from the heartburn frequency question*].
					Frequency	Categories	Before a year ago, how often did you have severe heartburn? x times per day/week/month/year, Don't know
					Duration	Continuous (granularity = months OR years = years)	Before a year ago, for how many months or years in total did you have severe heartburn? x number of months/years, Don't know
				Regurgitation	Recurrent / Non-recurrent	Categories	Before a year ago, did you ever have acid regurgitation, that is, a sour taste from contents of the stomach backing up into the mouth or throat? Yes, No, Don't know. [*Recurrent defined as at least once per week from the regurgitation frequency question*].
					Frequency	Categories	Before a year ago, how often did you have acid regurgitation? x times per day/week/month/year, Don't know
					Duration	Continuous (granularity = months OR years = years)	Before a year ago, for how many months or years in total did you experience acid regurgitation? x number of months/years, Don't know
Factors INfluencing the Barrett's Adenocarcinoma Relationship Study, Anderson, 2007	Ireland	2002–2004	5 years	Heartburn	Recurrent / Non-recurrent	Categories	Have you ever had frequent heartburn (a burning or ache behind the breastbone that is not due to heart problems) not including the last 5 years? Frequent means more than 50 time per year / about once per week. Yes, No, Don't know
					Frequency	Categories	How often did you suffer from heartburn when it was at it's most frequent (not including the last 5 years)? X times per day/week/month/year, Don't know
					Duration	Continuous (granularity = months AND years)	How long have you suffered from frequent heartburn (not including the last 5 years)? If a range is given, take the average. Years AND months.<br>Would you say from your: 20's, 30's, 40's, 50's, 60's, 70's and above, Don't know
				Regurgitation	Recurrent / Non-recurrent	Categories	Have you ever had frequent acid reflux i.e. a bitter taste of stomach acid which has come up to the back of your throat (not including the last 5 years)? Frequent means more than 50 time per year / about once per week. Yes. No, Don't know
					Frequency	Categories	How often did you suffer from acid reflux when it was at it's most frequent (not including the last 5 years)? X times per day/week/month/year, Don't know
					Duration	Continuous (granularity = months AND years)	How long have you suffered from frequent acid reflux (not including the last 5 years)? If a range is given, take the average. Years AND months.<br>Was your acid reflux most frequent during your: 20's, 30's, 40's, 50's, 60's, 70's and above, Don't know
Los Angeles County Multi-ethnic Case–control Study, Wu, 2001	United States	1992–1997	1 year	Heartburn	Recurrent / Non-recurrent	Categories	Before 19__, did you have heartburn after a meal (postprandial heartburn) excluding during pregnancy, this may move up and down your chest like a wave? Yes, No. [*Recurrent defined as weekly or daily from the heartburn frequency question*].
					Frequency	Categories	How often did you have heartburn after a meal (postprandial heartburn) excluding during pregnancy, this may move up and down your chest like a wave? Daily, weekly, monthly, <monthly
					Duration	Continuous (granularity = years)	How old were you (or in what year) did you first have heartburn after a meal (postprandial heartburn) excluding during pregnancy, this may move up and down your chest like a wave?
				Regurgitation	Recurrent / Non-recurrent	Categories	Before 19__, did you have sour stomach, acid indigestion, including regurgitation? Yes, No. [*Recurrent defined as weekly or daily from the regurgitation frequency question*].
					Frequency	Categories	How often did you have sour stomach, acid indigestion, including regurgitation? Daily, weekly, monthly, <monthly
					Duration	Continuous (granularity = years)	How old were you (or in what year) did you first have sour stomach, acid indigestion, including regurgitation?
Swedish Esophageal Cancer Study, Lagergren, 2000	Sweden	Dec 1, 1994 – Dec 31, 1997	5 years	Heartburn	Recurrent / Non-recurrent	Categories	Have you had frequent (in Swedish: often recurring) heartburn, not counting the previous 5 years? Frequent (often recurring) is defined as more than 50 times per year.
					Frequency	Continuous (granularity = days)	How often did you have heartburn (not counting previous 5 years)?
					Duration	Continuous (granularity = months)	For how long time were you troubled by heartburn (not counting previous 5 years)?
				Regurgitation	Recurrent / Non-recurrent	Categories	Have you had recurring acid regurgitations, i.e., sour taste of gastric acid coming up into your mouth (not counting previous 5 years)?
					Frequency	Continuous (granularity = days)	How often did you have acid regurgitations (not counting previous 5 years)?
					Duration	Continuous (granularity = months)	For how long did you have acid regurgitations (not counting previous 5 years)?

Supplementary Table 2. Associations between heartburn frequency, heartburn duration and esophageal adenocarcinoma.

		Heartburn Frequency		
		**Never**	**<weekly**	**≥weekly**
**Heartburn Duration (years)**	**Never**	*Referent*	-	-
	**0.1 to <20**	-	2.41 (95%CI:1.27-4.57; I2=49)	3.71 (95%CI:2.80-4.92; I2=23)
	**≥20**	-	2.65 (95%CI:1.63-4.31; I2=0)	7.74 (95%CI:3.96-15.15; I2=84)

Adjusted for age, sex, BMI, education, alcohol consumption, cigarette smoking, and study-specific variables.
Abbreviations: CI, confidence interval.

Supplementary Table 3. Associations between heartburn frequency, heartburn duration and esophagogastric junction adenocarcinoma.

		Heartburn Frequency		
		**Never**	**<weekly**	**≥weekly**
**Heartburn Duration (years)**	**Never**	*Referent*	-	-
	**0.1 to <20**	-	1.26 (95%CI:0.79-2.01; I2=0)	1.68 (95%CI:1.03-2.73; I2=71)
	**≥20**	-	1.64 (95%CI:0.95-2.86; I2=23)	3.38 (95%CI:1.79-6.41; I2=80)

Adjusted for age, sex, BMI, education, alcohol consumption, cigarette smoking, and study-specific variables.
Abbreviations: CI, confidence interval.

Supplementary Table 4. Associations between regurgitation frequency, regurgitation duration and esophageal adenocarcinoma.

		Regurgitation Frequency		
		**Never**	**<weekly**	**≥weekly**
**Regurgitation Duration (years)**	**Never**	*Referent*	-	-
	**0.1 to <20**	-	2.08 (95%CI:1.00-4.33; I2=71)	4.28 (95%CI:2.92-6.28; I2=54)
	**≥20**	-	2.31 (95%CI:0.96-5.58; I2=71)	6.43 (95%CI:4.01-10.30; I2=57)

Adjusted for age, sex, BMI, education, alcohol consumption, cigarette smoking, and study-specific variables.
Abbreviations: CI, confidence interval.

Supplementary Table 5. Associations between regurgitation frequency, regurgitation duration and esophagogastric junction adenocarcinoma.

		Regurgitation Frequency		
		**Never**	**<weekly**	**≥weekly**
**Regurgitation Duration (years)**	**Never**	*Referent*	-	-
	**0.1 to <20**	-	0.90 (95%CI:0.52-1.57; I2=44)	1.76 (95%CI:0.76-4.06; I2=89)
	**≥20**	-	1.48 (95%CI:0.58-3.78; I2=76)	2.01 (95%CI:1.11-3.64; I2=65)

Adjusted for age, sex, BMI, education, alcohol consumption, cigarette smoking, and study-specific variables.
Abbreviations: CI, confidence interval.

Supplementary Table 6. Associations between recurrent heartburn and/or recurrent regurgitation and adenocarcinomas of the esophagus in men.

Analysis	EA		EGJA	
	OR (95%CI)	I^2 (95%UI)	OR (95%CI)	I^2 (95%UI)
**Heartburn**				
Not recurrent	*Referent*		*Referent*	
Recurrent	4.33 (3.02-6.20)	71 (27-89)	2.33 (1.78-3.07)	46 (0-80)
**Regurgitation**				
Not recurrent	*Referent*		*Referent*	
Recurrent	4.40 (3.27-5.93)	52 (0-82)	1.86 (1.15-3.00)	78 (46-91)
**Heartburn or Regurgitation**				
Not recurrent	*Referent*		*Referent*	
Recurrent	4.65 (3.27-6.61)	73 (32-89)	2.30 (1.45-3.64)	83 (62-93)

Adjusted for age, BMI, education, alcohol consumption, cigarette smoking, and study-specific variables. Abbreviations: AA, all adenocarcinoma; CI, confidence interval; EA, esophgaeal adenocarcinoma; EGJA, esophagogastric junction adenocarcinoma; OR, odds ratio; UI, uncertainty interval.

Supplementary Table 7. Associations between heartburn and/or regurgitation duration and adenocarcinomas of the esophagus in men.

Analysis	EA		EGJA	
	OR (95%CI)	I^2 (95%UI)	OR (95%CI)	I^2 (95%UI)
**Heartburn Duration (years)**				
Never	*Referent*		*Referent*	
0.1 to <10	2.74 (1.66-4.53)	53 (0-83)	2.05 (1.36-3.07)	30 (0-73)
10 to <20	3.48 (2.59-4.68)	0 (0-12)	1.36 (0.69-2.67)	71 (27-89)
≥20	6.39 (3.47-11.80)	82 (59-92)	2.84 (1.54-5.26)	80 (54-92)
**Regurgitation Duration (years)**				
Never	*Referent*		*Referent*	
0.1 to <10	2.76 (1.44-5.29)	76 (41-90)	1.80 (0.73-4.45)	88 (75-94)
10 to <20	4.00 (2.29-7.00)	64 (4-86)	1.47 (0.69-3.11)	74 (36-90)
≥20	4.19 (2.24-7.82)	81 (56-92)	1.37 (0.69-2.75)	81 (56-92)
**Heartburn & Regurgitation Duration (years)**				
Never	*Referent*		*Referent*	
0.1 to <10	3.59 (1.54-8.41)	82 (57-92)	2.45 (0.79-7.64)	90 (79-95)
10 to <30	3.91 (2.26-6.78)	73 (32-89)	2.03 (1.08-3.81)	79 (51-91)
≥30	5.91 (3.27-10.68)	79 (49-91)	2.03 (1.07-3.85)	80 (52-91)

Adjusted for age, BMI, education, alcohol consumption, cigarette smoking, and study-specific variables. Abbreviations: AA, all adenocarcinoma; CI, confidence interval; EA, esophgaeal adenocarcinoma; EGJA, esophagogastric junction adenocarcinoma; OR, odds ratio; UI, uncertainty interval.

Supplementary Table 8. Associations between heartburn and/or regurgitation frequency and adenocarcinomas of the esophagus in men.

Analysis	EA		EGJA	
	OR (95%CI)	I^2 (95%UI)	OR (95%CI)	I^2 (95%UI)
**Heartburn Frequency**				
Never	*Referent*		*Referent*	
<monthly	0.90 (0.66-1.21)	0 (0-72)	0.61 (0.40-0.93)	36 (0-78)
Monthly to <weekly	3.00 (1.83-4.94)	51 (0-84)	1.83 (1.09-3.09)	55 (0-85)
Weekly to <daily	4.14 (2.75-6.24)	61 (0-85)	2.20 (1.45-3.35)	61 (0-86)
≥Daily	6.56 (3.53-12.19)	76 (42-90)	2.75 (1.92-3.93)	20 (0-83)
**Regurgitation Frequency**				
Never	*Referent*		*Referent*	
<monthly	0.68 (0.48-0.98)	23 (0-88)	0.58 (0.26-1.30)	86 (67-94)
Monthly to <weekly	3.16 (2.37-4.19)	0 (0-68)	1.67 (1.12-2.48)	39 (0-79)
Weekly to <daily	5.02 (3.40-7.41)	53 (0-83)	1.61 (0.74-3.49)	85 (68-93)
≥Daily	4.65 (2.88-7.50)	50 (0-82)	2.16 (1.53-3.06)	4 (0-80)
**Heartburn & Regurgitation Frequency**				
Never	*Referent*		*Referent*	
≤Weekly	1.96 (1.15-3.33)	79 (50-91)	1.25 (0.67-2.35)	88 (74-94)
>Weekly to Daily	5.21 (3.21-8.44)	68 (18-88)	2.17 (0.99-4.78)	87 (73-94)
>Daily	7.05 (3.82-13.00)	72 (31-89)	2.29 (1.55-3.37)	21 (0-66)

Adjusted for age, BMI, education, alcohol consumption, cigarette smoking, and study-specific variables. Abbreviations: AA, all adenocarcinoma; CI, confidence interval; EA, esophgaeal adenocarcinoma; EGJA, esophagogastric junction adenocarcinoma; OR, odds ratio; UI, uncertainty interval.

Supplementary Table 9. Associations between recurrent heartburn and/or recurrent regurgitation and adenocarcinomas of the esophagus in women.

Analysis	EA		EGJA	
	OR (95%CI)	I^2 (95%UI)	OR (95%CI)	I^2 (95%UI)
**Heartburn**				
Not recurrent	*Referent*		*Referent*	
Recurrent	11.68 (6.41-21.29)	0 (0-72)	3.30 (2.00-5.45)	0 (0-62)
**Regurgitation**				
Not recurrent	*Referent*		*Referent*	
Recurrent	7.81 (4.11-14.85)	0 (0-60)	2.01 (0.74-5.47)	58 (0-84)
**Heartburn or Regurgitation**				
Not recurrent	*Referent*		*Referent*	
Recurrent	10.09 (5.59-18.21)	0 (0-58)	2.70 (1.69-4.31)	0 (0-73)

Adjusted for age, BMI, education, alcohol consumption, cigarette smoking, and study-specific variables. Abbreviations: AA, all adenocarcinoma; CI, confidence interval; EA, esophgaeal adenocarcinoma; EGJA, esophagogastric junction adenocarcinoma; OR, odds ratio; UI, uncertainty interval.

Supplementary Table 10. Associations between heartburn and/or regurgitation duration and adenocarcinomas of the esophagus in women.

Analysis	EA		EGJA	
	OR (95%CI)	I^2 (95%UI)	OR (95%CI)	I^2 (95%UI)
**Heartburn Duration (years)**				
Never	*Referent*		*Referent*	
0.1 to <10	2.43 (0.53-11.26)	46 (0-82)	1.92 (0.82-4.53)	0 (0-63)
10 to <20	9.40 (2.64-33.46)	26 (0-72)	2.35 (0.26-21.23)	69 (0-91)
≥20	13.34 (5.65-31.50)	1 (0-85)	4.84 (2.41-9.68)	0 (0-73)
**Regurgitation Duration (years)**				
Never	*Referent*		*Referent*	
0.1 to <10	1.74 (0.65-4.63)	0 (0-84)	1.25 (0.14-11.02)	82 (55-93)
10 to <20	9.48 (2.62-34.27)	35 (0-78)	1.62 (0.42-6.26)	42 (0-80)
≥20	16.57 (5.95-46.20)	0 (0-0)	9.09 (3.66-22.60)	7 (0-86)
**Heartburn & Regurgitation Duration (years)**				
Never	*Referent*		*Referent*	
0.1 to <10	2.61 (0.27-25.03)	62 (0-89)	2.39 (0.37-15.49)	74 (26-91)
10 to <30	6.64 (2.42-18.22)	0 (0-84)	1.55 (0.37-6.49)	63 (0-88)
≥30	29.89 (10.40-85.93)	0 (0-75)	7.16 (2.38-21.53)	31 (0-75)

Adjusted for age, BMI, education, alcohol consumption, cigarette smoking, and study-specific variables. Abbreviations: AA, all adenocarcinoma; CI, confidence interval; EA, esophgaeal adenocarcinoma; EGJA, esophagogastric junction adenocarcinoma; OR, odds ratio; UI, uncertainty interval.

Supplementary Table 11. Associations between heartburn and/or regurgitation frequency and adenocarcinomas of the esophagus in women.

Analysis	EA		EGJA	
	OR (95%CI)	I^2 (95%UI)	OR (95%CI)	I^2 (95%UI)
**Heartburn Frequency**				
Never	*Referent*		*Referent*	
<monthly	0.94 (0.22-4.04)	36 (0-79)	0.38 (0.16-0.89)	0 (0-0)
Monthly to <weekly	1.30 (0.37-4.54)	0 (0-72)	1.34 (0.60-3.01)	0 (0-78)
Weekly to <daily	4.79 (1.90-12.11)	0 (0-0)	2.63 (1.05-6.60)	25 (0-71)
≥Daily	28.56 (7.35-110.90)	32 (0-76)	3.69 (1.32-10.33)	0 (0-83)
**Regurgitation Frequency**				
Never	*Referent*		*Referent*	
<monthly	1.27 (0.49-3.27)	0 (0-88)	1.15 (0.50-2.62)	0 (0-0)
Monthly to <weekly	2.27 (0.83-6.22)	0 (0-88)	2.37 (0.46-12.09)	71 (1-91)
Weekly to <daily	4.35 (1.61-11.70)	0 (0-85)	1.70 (0.26-11.18)	67 (0-90)
≥Daily	16.90 (2.86-99.97)	41 (0-80)	8.26 (2.86-23.86)	0 (0-86)
**Heartburn & Regurgitation Frequency**				
Never	*Referent*		*Referent*	
≤Weekly	1.21 (0.51-2.88)	0 (0-69)	0.86 (0.15-4.95)	72 (22-90)
>Weekly to Daily	3.54 (1.29-9.73)	0 (0-0)	2.73 (0.98-7.62)	0 (0-89)
>Daily	22.45 (7.74-65.10)	0 (0-70)	12.10 (4.17-35.07)	0 (0-29)

Adjusted for age, BMI, education, alcohol consumption, cigarette smoking, and study-specific variables. Abbreviations: AA, all adenocarcinoma; CI, confidence interval; EA, esophgaeal adenocarcinoma; EGJA, esophagogastric junction adenocarcinoma; OR, odds ratio; UI, uncertainty interval.

Supplementary Table 12. Associations between recurrent heartburn and/or recurrent regurgitation and adenocarcinomas of the esophagus in individuals with BMI<25.

Analysis	EA		EGJA	
	OR (95%CI)	I^2 (95%UI)	OR (95%CI)	I^2 (95%UI)
**Heartburn**				
Not recurrent	*Referent*		*Referent*	
Recurrent	4.77 (2.79-8.15)	66 (11-87)	2.45 (1.49-4.02)	58 (0-84)
**Regurgitation**				
Not recurrent	*Referent*		*Referent*	
Recurrent	5.20 (3.69-7.33)	7 (0-81)	1.79 (0.97-3.32)	63 (2-86)
**Heartburn or Regurgitation**				
Not recurrent	*Referent*		*Referent*	
Recurrent	4.70 (3.08-7.19)	52 (0-83)	2.14 (1.32-3.47)	62 (0-86)

Adjusted for age, sex, BMI (continuous), education, alcohol consumption, cigarette smoking, and study-specific variables. Abbreviations: AA, all adenocarcinoma; CI, confidence interval; EA, esophgaeal adenocarcinoma; EGJA, esophagogastric junction adenocarcinoma; OR, odds ratio; UI, uncertainty interval.

Supplementary Table 13. Associations between heartburn and/or regurgitation duration and adenocarcinomas of the esophagus in individuals with BMI<25.

Analysis	EA		EGJA	
	OR (95%CI)	I^2 (95%UI)	OR (95%CI)	I^2 (95%UI)
**Heartburn Duration (years)**				
Never	*Referent*		*Referent*	
0.1 to <10	2.26 (1.02-5.00)	40 (0-78)	2.05 (1.23-3.42)	0 (0-0)
10 to <20	3.64 (2.27-5.83)	0 (0-16)	0.82 (0.27-2.50)	64 (0-88)
≥20	6.26 (2.55-15.40)	77 (43-90)	3.87 (2.01-7.48)	58 (0-84)
**Regurgitation Duration (years)**				
Never	*Referent*		*Referent*	
0.1 to <10	2.44 (1.18-5.04)	51 (0-82)	1.31 (0.56-3.10)	65 (8-87)
10 to <20	6.77 (2.75-16.70)	63 (0-87)	1.31 (0.58-2.96)	39 (0-79)
≥20	4.55 (1.89-10.97)	73 (32-89)	2.57 (1.23-5.34)	66 (1-89)
**Heartburn & Regurgitation Duration (years)**				
Never	*Referent*		*Referent*	
0.1 to <10	2.74 (1.23-6.09)	41 (0-78)	2.06 (0.69-6.15)	72 (29-89)
10 to <30	4.52 (2.40-8.50)	46 (0-80)	1.68 (0.79-3.61)	61 (0-85)
≥30	6.34 (2.16-18.60)	81 (56-92)	3.19 (1.71-5.93)	53 (0-84)

Adjusted for age, sex, BMI (continuous), education, alcohol consumption, cigarette smoking, and study-specific variables. Abbreviations: AA, all adenocarcinoma; CI, confidence interval; EA, esophgaeal adenocarcinoma; EGJA, esophagogastric junction adenocarcinoma; OR, odds ratio; UI, uncertainty interval.

Supplementary Table 14. Associations between heartburn and/or regurgitation frequency and adenocarcinomas of the esophagus in individuals BMI<25.

Analysis	EA		EGJA	
	**OR (95%CI)**	***I*** ^{2} **(95%UI)**	**OR (95%CI)**	***I*** ^{2} **(95%UI)**
**Heartburn Frequency**				
Never	*Referent*		*Referent*	
<monthly	0.75 (0.43-1.31)	0 (0-30)	0.54 (0.29-0.98)	30 (0-93)
Monthly to <weekly	2.29 (1.12-4.67)	35 (0-78)	1.93 (1.09-3.41)	34 (0-77)
Weekly to <daily	4.58 (2.43-8.62)	58 (0-84)	2.41 (1.48-3.95)	41 (0-80)
≥Daily	6.78 (2.60-17.66)	74 (37-90)	2.70 (1.60-4.55)	0 (0-14)
**Regurgitation Frequency**				
Never	*Referent*		*Referent*	
<monthly	0.50 (0.24-1.05)	39 (0-81)	0.66 (0.24-1.79)	80 (46-92)
Monthly to <weekly	4.14 (2.34-7.32)	34 (0-77)	1.92 (1.23-3.00)	0 (0-18)
Weekly to <daily	4.98 (3.21-7.72)	8 (0-81)	1.73 (0.87-3.41)	54 (0-85)
≥Daily	7.10 (3.91-12.89)	14 (0-82)	2.35 (1.34-4.12)	0 (0-73)
**Heartburn & Regurgitation Frequency**				
Never	*Referent*		*Referent*	
≤Weekly	1.85 (1.14-3.01)	39 (0-77)	1.07 (0.63-1.82)	69 (9-89)
>Weekly to Daily	5.55 (2.91-10.58)	52 (0-82)	2.41 (1.33-4.39)	47 (0-81)
>Daily	7.75 (2.97-20.19)	69 (19-88)	2.08 (1.16-3.73)	6 (0-80)

Adjusted for age, sex, BMI (continuous), education, alcohol consumption, cigarette smoking, and study-specific variables. Abbreviations: AA, all adenocarcinoma; CI, confidence interval; EA, esophgaeal adenocarcinoma; EGJA, esophagogastric junction adenocarcinoma; OR, odds ratio; UI, uncertainty interval.

Supplementary Table 15. Associations between recurrent heartburn and/or recurrent regurgitation and adenocarcinomas of the esophagus in individuals with BMI≥25 & <30.

Analysis	EA		EGJA	
	**OR (95%CI)**	***I*** ^{2} **(95%UI)**	**OR (95%CI)**	***I*** ^{2} **(95%UI)**
**Heartburn**				
Not recurrent	*Referent*		*Referent*	
Recurrent	4.56 (2.66-7.82)	71 (28-89)	2.53 (1.61-4.00)	55 (0-84)
**Regurgitation**				
Not recurrent	*Referent*		*Referent*	
Recurrent	4.58 (2.77-7.57)	62 (0-86)	2.12 (0.97-4.62)	81 (56-92)
**Heartburn or Regurgitation**				
Not recurrent	*Referent*		*Referent*	
Recurrent	5.23 (3.04-9.02)	74 (36-90)	2.57 (1.25-5.29)	85 (65-93)

Adjusted for age, sex, BMI (continuous), education, alcohol consumption, cigarette smoking, and study-specific variables. Abbreviations: AA, all adenocarcinoma; CI, confidence interval; EA, esophgaeal adenocarcinoma; EGJA, esophagogastric junction adenocarcinoma; OR, odds ratio; UI, uncertainty interval.

Supplementary Table 16. Associations between heartburn and/or regurgitation duration and adenocarcinomas of the esophagus in individuals with BMI≥25 & <30.

Analysis	EA		EGJA	
	OR (95%CI)	I^2 (95%UI)	OR (95%CI)	I^2 (95%UI)
**Heartburn Duration (years)**				
Never	*Referent*		*Referent*	
0.1 to <10	3.20 (1.47-6.98)	58 (0-84)	2.19 (0.90-5.34)	67 (13-87)
10 to <20	3.67 (2.34-5.74)	0 (0-3)	2.07 (1.00-4.27)	46 (0-80)
≥20	7.00 (3.26-15.06)	74 (34-89)	2.34 (0.93-5.89)	77 (46-91)
**Regurgitation Duration (years)**				
Never	*Referent*		*Referent*	
0.1 to <10	2.68 (1.62-4.43)	20 (0-83)	1.99 (0.57-6.87)	86 (70-94)
10 to <20	4.58 (2.72-7.73)	18 (0-83)	1.61 (0.58-4.42)	68 (18-88)
≥20	4.99 (2.35-10.60)	68 (19-88)	1.56 (0.41-5.87)	87 (71-94)
**Heartburn & Regurgitation Duration (years)**				
Never	*Referent*		*Referent*	
0.1 to <10	3.57 (1.38-9.23)	66 (12-87)	3.12 (0.48-20.22)	91 (82-96)
10 to <30	4.54 (2.66-7.75)	39 (0-77)	2.16 (0.77-6.06)	82 (59-92)
≥30	6.11 (3.04-12.29)	65 (9-87)	1.92 (0.62-5.89)	83 (61-93)

Adjusted for age, sex, BMI (continuous), education, alcohol consumption, cigarette smoking, and study-specific variables. Abbreviations: AA, all adenocarcinoma; CI, confidence interval; EA, esophgaeal adenocarcinoma; EGJA, esophagogastric junction adenocarcinoma; OR, odds ratio; UI, uncertainty interval.

Supplementary Table 17. Associations between heartburn and/or regurgitation frequency and adenocarcinomas of the esophagus in individuals BMI≥25 & <30.

Analysis	EA		EGJA	
	OR (95%CI)	I^2 (95%UI)	OR (95%CI)	I^2 (95%UI)
**Heartburn Frequency**				
Never	*Referent*		*Referent*	
<monthly	1.16 (0.77-1.75)	0 (0-51)	0.50 (0.32-0.79)	0 (0-75)
Monthly to <weekly	2.59 (1.64-4.07)	0 (0-84)	2.05 (1.14-3.70)	23 (0-92)
Weekly to <daily	4.98 (2.96-8.39)	47 (0-80)	2.38 (1.18-4.82)	68 (17-88)
≥Daily	5.78 (2.93-11.40)	54 (0-83)	3.11 (1.41-6.87)	62 (0-86)
**Regurgitation Frequency**				
Never	*Referent*		*Referent*	
<monthly	0.86 (0.49-1.52)	37 (0-78)	0.44 (0.16-1.24)	77 (37-92)
Monthly to <weekly	2.87 (1.70-4.84)	28 (0-73)	1.84 (1.04-3.27)	32 (0-76)
Weekly to <daily	6.06 (3.64-10.11)	39 (0-77)	1.95 (0.66-5.75)	83 (62-93)
≥Daily	3.76 (2.01-7.04)	33 (0-75)	2.23 (0.82-6.06)	71 (25-88)
**Heartburn & Regurgitation Frequency**				
Never	*Referent*		*Referent*	
≤Weekly	2.20 (1.10-4.39)	72 (30-89)	1.14 (0.42-3.05)	86 (68-93)
>Weekly to Daily	5.87 (3.16-10.93)	56 (0-84)	2.36 (0.71-7.85)	87 (72-94)
>Daily	6.66 (3.36-13.18)	48 (0-81)	3.16 (1.17-8.55)	71 (26-89)

Adjusted for age, sex, BMI (continuous), education, alcohol consumption, cigarette smoking, and study-specific variables. Abbreviations: AA, all adenocarcinoma; CI, confidence interval; EA, esophgaeal adenocarcinoma; EGJA, esophagogastric junction adenocarcinoma; OR, odds ratio; UI, uncertainty interval.

Supplementary Table 18. Associations between recurrent heartburn and/or recurrent regurgitation and adenocarcinomas of the esophagus in individuals with BMI≥30.

Analysis	EA		EGJA	
	OR (95%CI)	I^2 (95%UI)	OR (95%CI)	I^2 (95%UI)
**Heartburn**				
Not recurrent	*Referent*		*Referent*	
Recurrent	5.55 (3.45-8.92)	0 (0-47)	2.67 (1.72-4.14)	0 (0-77)
**Regurgitation**				
Not recurrent	*Referent*		*Referent*	
Recurrent	5.71 (3.54-9.23)	0 (0-78)	2.28 (1.44-3.61)	0 (0-76)
**Heartburn or Regurgitation**				
Not recurrent	*Referent*		*Referent*	
Recurrent	5.69 (3.38-9.58)	12 (0-87)	2.86 (1.91-4.27)	0 (0-52)

Adjusted for age, sex, BMI (continuous), education, alcohol consumption, cigarette smoking, and study-specific variables. Abbreviations: AA, all adenocarcinoma; CI, confidence interval; EA, esophgaeal adenocarcinoma; EGJA, esophagogastric junction adenocarcinoma; OR, odds ratio; UI, uncertainty interval.

Supplementary Table 19. Associations between heartburn and/or regurgitation duration and adenocarcinomas of the esophagus in individuals with BMI≥30.

Analysis	EA		EGJA	
	OR (95%CI)	I^2 (95%UI)	OR (95%CI)	I^2 (95%UI)
**Heartburn Duration (years)**				
Never	*Referent*		*Referent*	
0.1 to <10	3.67 (0.92-14.66)	53 (0-84)	0.87 (0.17-4.35)	53 (0-86)
10 to <20	6.61 (2.73-15.97)	11 (0-86)	2.36 (0.28-19.58)	73 (11-92)
≥20	6.14 (1.46-25.80)	73 (8-92)	4.27 (2.28-8.01)	0 (0-68)
**Regurgitation Duration (years)**				
Never	*Referent*		*Referent*	
0.1 to <10	3.55 (0.89-14.15)	46 (0-84)	2.54 (0.49-13.10)	48 (0-85)
10 to <20	5.60 (2.25-13.92)	0 (0-52)	4.04 (1.52-10.74)	10 (0-81)
≥20	5.52 (2.69-11.30)	6 (0-80)	3.51 (1.62-7.58)	0 (0-67)
**Heartburn & Regurgitation Duration (years)**				
Never	*Referent*		*Referent*	
0.1 to <10	3.65 (0.20-65.20)	78 (30-93)	3.16 (0.60-16.72)	47 (0-83)
10 to <30	5.87 (2.42-14.23)	0 (0-78)	6.38 (1.18-34.41)	61 (0-85)
≥30	6.75 (1.64-27.78)	67 (5-89)	6.17 (2.74-13.88)	0 (0-46)

Adjusted for age, sex, BMI (continuous), education, alcohol consumption, cigarette smoking, and study-specific variables. Abbreviations: AA, all adenocarcinoma; CI, confidence interval; EA, esophgaeal adenocarcinoma; EGJA, esophagogastric junction adenocarcinoma; OR, odds ratio; UI, uncertainty interval.

Supplementary Table 20. Associations between heartburn and/or regurgitation frequency and adenocarcinomas of the esophagus in individuals BMI≥30.

Analysis	EA		EGJA	
	OR (95%CI)	I^2 (95%UI)	OR (95%CI)	I^2 (95%UI)
**Heartburn Frequency**				
Never	*Referent*		*Referent*	
<monthly	0.50 (0.21-1.19)	13 (0-91)	0.77 (0.45-1.33)	0 (0-0)
Monthly to <weekly	3.50 (1.13-10.80)	50 (0-87)	1.54 (0.82-2.91)	0 (0-0)
Weekly to <daily	3.58 (1.06-12.04)	55 (0-87)	2.95 (1.64-5.30)	0 (0-64)
≥Daily	10.10 (4.38-23.26)	24 (0-92)	2.57 (1.25-5.25)	0 (0-0)
**Regurgitation Frequency**				
Never	*Referent*		*Referent*	
<monthly	0.79 (0.16-4.02)	71 (0-91)	1.14 (0.45-2.90)	36 (0-79)
Monthly to <weekly	1.75 (0.19-16.39)	77 (1-95)	1.86 (1.00-3.47)	0 (0-82)
Weekly to <daily	4.62 (1.47-14.50)	44 (0-79)	2.74 (1.16-6.48)	22 (0-67)
≥Daily	5.33 (2.63-10.78)	0 (0-78)	1.83 (0.49-6.80)	51 (0-84)
**Heartburn & Regurgitation Frequency**				
Never	*Referent*		*Referent*	
≤Weekly	1.94 (0.37-10.03)	75 (39-90)	2.24 (0.68-7.34)	64 (0-88)
>Weekly to Daily	3.11 (0.36-26.65)	83 (48-94)	3.27 (1.71-6.27)	0 (0-74)
>Daily	7.42 (3.26-16.89)	24 (0-88)	2.47 (1.19-5.13)	0 (0-30)

Adjusted for age, sex, BMI (continuous), education, alcohol consumption, cigarette smoking, and study-specific variables. Abbreviations: AA, all adenocarcinoma; CI, confidence interval; EA, esophgaeal adenocarcinoma; EGJA, esophagogastric junction adenocarcinoma; OR, odds ratio; UI, uncertainty interval.

Supplementary Table 21. Associations between recurrent heartburn and/or recurrent regurgitation and adenocarcinomas of the esophagus in individuals <60 years of age at diagnosis/recruitment.

**Analysis**	**EA**		**EGJA**	
	**OR (95%CI)**	***I*** ^{2} **(95%UI)**	**OR (95%CI)**	***I*** ^{2} **(95%UI)**
**Heartburn**				
Not recurrent	*Referent*		*Referent*	
Recurrent	3.88 (2.51-6.00)	38 (0-77)	2.68 (1.85-3.90)	21 (0-66)
**Regurgitation**				
Not recurrent	*Referent*		*Referent*	
Recurrent	3.71 (2.21-6.22)	50 (0-82)	1.92 (1.14-3.26)	48 (0-81)
**Heartburn or Regurgitation**				
Not recurrent	*Referent*		*Referent*	
Recurrent	4.01 (2.57-6.25)	46 (0-80)	2.31 (1.45-3.67)	53 (0-83)

Adjusted for age, sex, BMI, education, alcohol consumption, cigarette smoking, and study-specific variables. Abbreviations: AA, all adenocarcinoma; CI, confidence interval; EA, esophgaeal adenocarcinoma; EGJA, esophagogastric junction adenocarcinoma; OR, odds ratio; UI, uncertainty interval.

Supplementary Table 22. Associations between heartburn and/or regurgitation duration and adenocarcinomas of the esophagus in individuals <60 years of age at diagnosis/recruitment.

Analysis	EA		EGJA	
	OR (95%CI)	I^2 (95%UI)	OR (95%CI)	I^2 (95%UI)
**Heartburn Duration (years)**				
Never	*Referent*		*Referent*	
0.1 to <10	1.62 (0.65-4.08)	61 (0-87)	1.72 (0.90-3.26)	36 (0-76)
10 to <20	3.75 (2.11-6.66)	14 (0-82)	1.46 (0.61-3.49)	46 (0-80)
≥20	5.00 (2.49-10.04)	52 (0-82)	3.14 (1.24-7.92)	74 (35-89)
**Regurgitation Duration (years)**				
Never	*Referent*		*Referent*	
0.1 to <10	3.02 (0.97-9.39)	77 (45-91)	1.46 (0.44-4.88)	81 (56-92)
10 to <20	4.16 (1.61-10.75)	58 (0-84)	1.25 (0.25-6.19)	82 (54-93)
≥20	3.13 (0.99-9.88)	79 (51-91)	0.99 (0.31-3.11)	76 (42-90)
**Heartburn & Regurgitation Duration (years)**				
Never	*Referent*		*Referent*	
0.1 to <10	3.06 (0.82-11.36)	76 (41-90)	1.82 (0.48-6.99)	80 (54-92)
10 to <30	3.17 (1.31-7.66)	65 (9-87)	1.90 (0.53-6.84)	84 (65-93)
≥30	4.51 (1.83-11.08)	68 (16-88)	1.66 (0.63-4.35)	70 (23-88)

Adjusted for age, sex, BMI, education, alcohol consumption, cigarette smoking, and study-specific variables. Abbreviations: AA, all adenocarcinoma; CI, confidence interval; EA, esophgaeal adenocarcinoma; EGJA, esophagogastric junction adenocarcinoma; OR, odds ratio; UI, uncertainty interval.

Supplementary Table 23. Associations between heartburn and/or regurgitation frequency and adenocarcinomas of the esophagus in individuals <60 years of age at diagnosis/recruitment.

Analysis	EA		EGJA	
	**OR (95%CI)**	**I^2 (95%UI)**	**OR (95%CI)**	**I^2 (95%UI)**
**Heartburn Frequency**				
Never	*Referent*		*Referent*	
<monthly	0.75 (0.46-1.24)	0 (0-68)	0.63 (0.33-1.23)	36 (0-78)
Monthly to <weekly	2.22 (1.31-3.75)	0 (0-6)	1.86 (1.15-2.99)	0 (0-36)
Weekly to <daily	3.19 (1.60-6.33)	54 (0-83)	2.28 (1.19-4.36)	54 (0-83)
≥Daily	5.22 (3.15-8.67)	2 (0-80)	2.94 (1.74-4.95)	0 (0-63)
**Regurgitation Frequency**				
Never	*Referent*		*Referent*	
<monthly	0.56 (0.19-1.62)	74 (15-92)	0.33 (0.11-0.98)	74 (27-91)
Monthly to <weekly	3.31 (1.94-5.64)	3 (0-85)	1.40 (0.70-2.78)	35 (0-77)
Weekly to <daily	3.87 (1.81-8.30)	60 (0-85)	1.01 (0.41-2.46)	65 (10-87)
≥Daily	4.21 (2.06-8.60)	24 (0-69)	3.31 (1.86-5.89)	0 (0-65)
**Heartburn & Regurgitation Frequency**				
Never	*Referent*		*Referent*	
≤Weekly	1.36 (0.79-2.36)	39 (0-77)	1.11 (0.43-2.86)	83 (62-93)
>Weekly to Daily	3.44 (1.50-7.87)	64 (4-86)	1.76 (0.76-4.05)	66 (10-87)
>Daily	5.41 (2.84-10.32)	26 (0-71)	2.77 (1.53-5.02)	11 (0-82)

Adjusted for age, sex, BMI, education, alcohol consumption, cigarette smoking, and study-specific variables. Abbreviations: AA, all adenocarcinoma; CI, confidence interval; EA, esophgaeal adenocarcinoma; EGJA, esophagogastric junction adenocarcinoma; OR, odds ratio; UI, uncertainty interval.

Supplementary Table 24. Associations between recurrent heartburn and/or recurrent regurgitation and adenocarcinomas of the esophagus in individuals ≥60 and <70 years of age at diagnosis/recruitment.

Analysis	EA		EGJA	
	**OR (95%CI)**	***I^2* (95%UI)**	**OR (95%CI)**	***I^2* (95%UI)**
**Heartburn**				
Not recurrent	*Referent*		*Referent*	
Recurrent	6.57 (3.87-11.15)	60 (0-85)	2.21 (1.62-3.00)	0 (0-0)
**Regurgitation**				
Not recurrent	*Referent*		*Referent*	
Recurrent	5.12 (3.72-7.05)	0 (0-62)	1.85 (0.99-3.49)	66 (10-87)
**Heartburn or Regurgitation**				
Not recurrent	*Referent*		*Referent*	
Recurrent	6.03 (4.50-8.07)	0 (0-78)	2.49 (1.68-3.70)	42 (0-79)

Adjusted for age, sex, BMI, education, alcohol consumption, cigarette smoking, and study-specific variables. Abbreviations: AA, all adenocarcinoma; CI, confidence interval; EA, esophgaeal adenocarcinoma; EGJA, esophagogastric junction adenocarcinoma; OR, odds ratio; UI, uncertainty interval.

Supplementary Table 25. Associations between heartburn and/or regurgitation duration and adenocarcinomas of the esophagus in individuals ≥60 and <70 years of age at diagnosis/recruitment.

Analysis	EA		EGJA	
	OR (95%CI)	I^2 (95%UI)	OR (95%CI)	I^2 (95%UI)
**Heartburn Duration (years)**				
Never	*Referent*		*Referent*	
0.1 to <10	4.75 (1.93-11.66)	48 (0-81)	2.28 (1.29-4.02)	0 (0-0)
10 to <20	6.13 (3.82-9.85)	0 (0-54)	1.61 (0.95-2.71)	0 (0-51)
≥20	10.33 (4.22-25.27)	70 (23-88)	3.41 (1.49-7.79)	67 (14-87)
**Regurgitation Duration (years)**				
Never	*Referent*		*Referent*	
0.1 to <10	3.01 (1.83-4.95)	0 (0-73)	2.12 (0.74-6.07)	79 (49-91)
10 to <20	3.91 (2.30-6.65)	3 (0-80)	1.49 (0.61-3.66)	57 (0-84)
≥20	5.57 (3.41-9.10)	13 (0-82)	2.38 (1.23-4.62)	45 (0-80)
**Heartburn & Regurgitation Duration (years)**				
Never	*Referent*		*Referent*	
0.1 to <10	4.52 (1.97-10.38)	41 (0-80)	2.36 (0.61-9.10)	81 (50-93)
10 to <30	6.18 (3.87-9.87)	0 (0-41)	2.11 (1.14-3.88)	44 (0-79)
≥30	9.33 (4.98-17.48)	37 (0-76)	2.84 (1.49-5.43)	46 (0-80)

Adjusted for age, sex, BMI, education, alcohol consumption, cigarette smoking, and study-specific variables. Abbreviations: AA, all adenocarcinoma; CI, confidence interval; EA, esophgaeal adenocarcinoma; EGJA, esophagogastric junction adenocarcinoma; OR, odds ratio; UI, uncertainty interval.

Supplementary Table 26. Associations between heartburn and/or regurgitation frequency and adenocarcinomas of the esophagus in individuals ≥60 and <70 years of age at diagnosis/recruitment.

Analysis	EA		EGJA	
	**OR (95%CI)**	**I^2 (95%UI)**	**OR (95%CI)**	**I^2 (95%UI)**
**Heartburn Frequency**				
Never	*Referent*		*Referent*	
<monthly	0.95 (0.57-1.58)	0 (0-62)	0.45 (0.28-0.73)	0 (0-85)
Monthly to <weekly	3.88 (2.30-6.54)	2 (0-85)	1.78 (1.08-2.92)	4 (0-85)
Weekly to <daily	6.41 (4.00-10.28)	20 (0-83)	2.14 (1.44-3.18)	0 (0-46)
≥Daily	10.95 (4.91-24.44)	56 (0-84)	2.11 (1.24-3.58)	0 (0-73)
**Regurgitation Frequency**				
Never	*Referent*		*Referent*	
<monthly	0.75 (0.46-1.22)	0 (0-0)	0.80 (0.36-1.79)	65 (0-88)
Monthly to <weekly	2.83 (1.81-4.43)	0 (0-77)	2.12 (1.38-3.23)	0 (0-68)
Weekly to <daily	6.72 (4.43-10.22)	0 (0-58)	2.09 (0.80-5.51)	75 (37-90)
≥Daily	5.18 (3.02-8.89)	0 (0-71)	2.79 (1.58-4.92)	0 (0-83)
**Heartburn & Regurgitation Frequency**				
Never	*Referent*		*Referent*	
≤Weekly	2.42 (1.19-4.94)	67 (3-89)	1.30 (0.68-2.50)	73 (24-90)
>Weekly to Daily	8.82 (5.60-13.89)	0 (0-54)	2.29 (1.09-4.80)	60 (0-85)
>Daily	11.91 (4.75-29.85)	58 (0-84)	2.51 (1.44-4.37)	0 (0-60)

Adjusted for age, sex, BMI, education, alcohol consumption, cigarette smoking, and study-specific variables. Abbreviations: AA, all adenocarcinoma; CI, confidence interval; EA, esophgaeal adenocarcinoma; EGJA, esophagogastric junction adenocarcinoma; OR, odds ratio; UI, uncertainty interval.

Supplementary Table 27. Associations between recurrent heartburn and/or recurrent regurgitation and adenocarcinomas of the esophagus in individuals ≥70 years of age at diagnosis/recruitment.

Analysis	EA		EGJA	
	OR (95%CI)	I^2 (95%UI)	OR (95%CI)	I^2 (95%UI)
**Heartburn**				
Not recurrent	*Referent*		*Referent*	
Recurrent	5.68 (3.52-9.17)	51 (0-82)	2.93 (1.78-4.83)	50 (0-82)
**Regurgitation**				
Not recurrent	*Referent*		*Referent*	
Recurrent	5.97 (4.15-8.59)	3 (0-80)	2.26 (1.36-3.76)	36 (0-76)
**Heartburn or Regurgitation**				
Not recurrent	*Referent*		*Referent*	
Recurrent	5.69 (3.57-9.06)	54 (0-83)	2.47 (1.38-4.44)	68 (17-88)

Adjusted for age, sex, BMI, education, alcohol consumption, cigarette smoking, and study-specific variables. Abbreviations: AA, all adenocarcinoma; CI, confidence interval; EA, esophgaeal adenocarcinoma; EGJA, esophagogastric junction adenocarcinoma; OR, odds ratio; UI, uncertainty interval.

Supplementary Table 28. Associations between heartburn and/or regurgitation duration and adenocarcinomas of the esophagus in individuals ≥70 years of age at diagnosis/recruitment.

Analysis	EA		EGJA	
	OR (95%CI)	I^2 (95%UI)	OR (95%CI)	I^2 (95%UI)
**Heartburn Duration (years)**				
Never	*Referent*		*Referent*	
0.1 to <10	4.03 (2.20-7.37)	0 (0-70)	2.75 (0.79-9.54)	67 (15-87)
10 to <20	2.94 (1.70-5.09)	0 (0-55)	2.21 (1.03-4.74)	0 (0-78)
≥20	7.46 (3.30-16.87)	72 (28-89)	2.50 (1.12-5.56)	64 (4-86)
**Regurgitation Duration (years)**				
Never	*Referent*		*Referent*	
0.1 to <10	2.43 (1.00-5.94)	51 (0-82)	2.09 (0.47-9.21)	81 (57-92)
10 to <20	5.67 (2.83-11.39)	33 (0-74)	1.50 (0.74-3.04)	0 (0-24)
≥20	6.44 (3.46-11.98)	43 (0-79)	1.73 (0.65-4.60)	68 (18-88)
**Heartburn & Regurgitation Duration (years)**				
Never	*Referent*		*Referent*	
0.1 to <10	2.74 (1.30-5.80)	28 (0-72)	2.67 (0.58-12.28)	80 (53-92)
10 to <30	4.17 (2.57-6.78)	8 (0-81)	1.78 (0.99-3.20)	19 (0-83)
≥30	8.27 (3.73-18.36)	65 (7-87)	2.14 (0.75-6.14)	74 (34-89)

Adjusted for age, sex, BMI, education, alcohol consumption, cigarette smoking, and study-specific variables. Abbreviations: AA, all adenocarcinoma; CI, confidence interval; EA, esophgaeal adenocarcinoma; EGJA, esophagogastric junction adenocarcinoma; OR, odds ratio; UI, uncertainty interval.

Supplementary Table 29. Associations between heartburn and/or regurgitation frequency and adenocarcinomas of the esophagus in individuals ≥70 years of age at diagnosis/recruitment.

Analysis	EA		EGJA	
	OR (95%CI)	I^2 (95%UI)	OR (95%CI)	I^2 (95%UI)
**Heartburn Frequency**				
Never	*Referent*		*Referent*	
<monthly	0.90 (0.33-2.46)	61 (0-89)	0.64 (0.17-2.46)	78 (28-93)
Monthly to <weekly	3.44 (1.29-9.15)	61 (0-89)	1.81 (1.04-3.14)	0 (0-0)
Weekly to <daily	5.18 (2.70-9.96)	55 (0-83)	2.18 (1.14-4.17)	50 (0-82)
≥Daily	11.11 (4.81-25.70)	54 (0-83)	6.07 (2.34-15.75)	56 (0-84)
**Regurgitation Frequency**				
Never	*Referent*		*Referent*	
<monthly	0.81 (0.47-1.38)	0 (0-79)	0.66 (0.25-1.74)	68 (6-89)
Monthly to <weekly	3.83 (2.03-7.24)	33 (0-75)	1.72 (0.66-4.45)	64 (0-88)
Weekly to <daily	7.08 (4.49-11.14)	0 (0-60)	2.32 (1.09-4.91)	51 (0-82)
≥Daily	5.82 (2.31-14.69)	56 (0-84)	2.33 (0.97-5.61)	45 (0-80)
**Heartburn & Regurgitation Frequency**				
Never	*Referent*		*Referent*	
≤Weekly	1.87 (0.79-4.39)	78 (48-91)	0.98 (0.43-2.23)	76 (40-90)
>Weekly to Daily	6.71 (3.38-13.32)	53 (0-83)	2.71 (1.18-6.25)	64 (7-86)
>Daily	11.13 (3.78-32.78)	70 (23-88)	3.92 (1.13-13.62)	72 (29-89)

Adjusted for age, sex, BMI, education, alcohol consumption, cigarette smoking, and study-specific variables. Abbreviations: AA, all adenocarcinoma; CI, confidence interval; EA, esophgaeal adenocarcinoma; EGJA, esophagogastric junction adenocarcinoma; OR, odds ratio; UI, uncertainty interval.

Supplementary Table 30. Associations between recurrent heartburn and/or recurrent regurgitation and adenocarcinomas of the esophagus in never-cigarette smokers.

Analysis	EA		EGJA	
	OR (95%CI)	I^2 (95%UI)	OR (95%CI)	I^2 (95%UI)
**Heartburn**				
Not recurrent	*Referent*		*Referent*	
Recurrent	5.34 (3.44-8.30)	30 (0-73)	2.28 (1.55-3.33)	0 (0-3)
**Regurgitation**				
Not recurrent	*Referent*		*Referent*	
Recurrent	4.88 (2.93-8.14)	42 (0-78)	1.76 (0.73-4.24)	70 (23-4)
**Heartburn or Regurgitation**				
Not recurrent	*Referent*		*Referent*	
Recurrent	5.73 (3.81-8.62)	28 (0-72)	2.13 (1.33-3.42)	36 (0-3)

Adjusted for age, sex, BMI, education, alcohol consumption, and study-specific variables. Abbreviations: AA, all adenocarcinoma; CI, confidence interval; EA, esophgaeal adenocarcinoma; EGJA, esophagogastric junction adenocarcinoma; OR, odds ratio; UI, uncertainty interval.

Supplementary Table 31. Associations between heartburn and/or regurgitation duration and adenocarcinomas of the esophagus never-cigarette smokers.

Analysis	EA		EGJA	
	OR (95%CI)	I^2 (95%UI)	OR (95%CI)	I^2 (95%UI)
**Heartburn Duration (years)**				
Never	*Referent*		*Referent*	
0.1 to <10	3.03 (1.22-7.51)	51 (0-84)	0.96 (0.43-2.12)	0 (0-81)
10 to <20	3.32 (1.47-7.49)	35 (0-77)	1.47 (0.47-4.62)	55 (0-85)
≥20	7.66 (2.30-25.51)	80 (46-92)	2.84 (1.12-7.21)	62 (0-87)
**Regurgitation Duration (years)**				
Never	*Referent*		*Referent*	
0.1 to <10	2.65 (1.27-5.52)	33 (0-76)	1.55 (0.37-6.44)	79 (45-92)
10 to <20	5.72 (1.74-18.84)	71 (18-90)	1.48 (0.35-6.35)	68 (7-89)
≥20	3.88 (1.52-9.94)	67 (3-89)	3.21 (0.99-10.38)	72 (5-92)
**Heartburn & Regurgitation Duration (years)**				
Never	*Referent*		*Referent*	
0.1 to <10	3.59 (1.59-8.14)	33 (0-77)	1.43 (0.34-5.98)	71 (19-90)
10 to <30	5.00 (2.78-8.98)	13 (0-87)	1.46 (0.38-5.68)	78 (41-92)
≥30	7.62 (3.13-18.57)	60 (0-87)	2.05 (0.63-6.65)	73 (25-91)

Adjusted for age, sex, BMI, education, alcohol consumption, and study-specific variables.
Abbreviations: AA, all adenocarcinoma; CI, confidence interval; EA, esophgaeal adenocarcinoma; EGJA, esophagogastric junction adenocarcinoma; OR, odds ratio; UI, uncertainty interval.

Supplementary Table 32. Associations between heartburn and/or regurgitation frequency and adenocarcinomas of the esophagus never-cigarette smokers.

Analysis	EA		EGJA	
	OR (95%CI)	I^2 (95%UI)	OR (95%CI)	I^2 (95%UI)
**Heartburn Frequency**				
Never	*Referent*		*Referent*	
<monthly	0.89 (0.49-1.62)	0 (0-0)	0.54 (0.29-1.00)	3 (0-90)
Monthly to <weekly	3.85 (2.19-6.77)	0 (0-80)	2.12 (1.19-3.77)	0 (0-33)
Weekly to <daily	5.25 (3.00-9.19)	30 (0-73)	1.60 (0.84-3.05)	28 (0-72)
≥Daily	11.52 (5.75-23.06)	25 (0-70)	3.91 (2.09-7.31)	0 (0-70)
**Regurgitation Frequency**				
Never	*Referent*		*Referent*	
<monthly	0.70 (0.41-1.20)	0 (0-37)	0.98 (0.59-1.63)	0 (0-71)
Monthly to <weekly	3.30 (1.49-7.33)	49 (0-83)	2.11 (0.85-5.21)	53 (0-84)
Weekly to <daily	5.40 (2.83-10.29)	44 (0-80)	1.40 (0.32-6.25)	82 (58-92)
≥Daily	6.07 (2.81-13.13)	23 (0-88)	2.48 (1.12-5.50)	0 (0-56)
**Heartburn & Regurgitation Frequency**				
Never	*Referent*		*Referent*	
≤Weekly	2.50 (1.20-5.20)	64 (5-86)	0.94 (0.45-1.97)	59 (0-85)
>Weekly to Daily	6.49 (3.62-11.65)	20 (0-83)	2.26 (0.93-5.47)	58 (0-84)
>Daily	12.81 (5.58-29.43)	41 (0-78)	4.03 (1.54-10.55)	36 (0-76)

Adjusted for age, sex, BMI, education, alcohol consumption, and study-specific variables.
Abbreviations: AA, all adenocarcinoma; CI, confidence interval; EA, esophgaeal adenocarcinoma; EGJA, esophagogastric junction adenocarcinoma; OR, odds ratio; UI, uncertainty interval.

Supplementary Table 33. Associations between recurrent heartburn and/or recurrent regurgitation and adenocarcinomas of the esophagus in ever-cigarette smokers.

Analysis	EA		EGJA	
	OR (95%CI)	I^2 (95%UI)	OR (95%CI)	I^2 (95%UI)
**Heartburn**				
Not recurrent	*Referent*		*Referent*	
Recurrent	4.61 (3.14-6.78)	69 (21-88)	2.55 (1.95-3.34)	36 (0-76)
**Regurgitation**				
Not recurrent	*Referent*		*Referent*	
Recurrent	4.51 (3.40-5.99)	36 (0-76)	2.00 (1.22-3.25)	76 (41-90)
**Heartburn or Regurgitation**				
Not recurrent	*Referent*		*Referent*	
Recurrent	4.70 (3.20-6.92)	73 (31-89)	2.44 (1.51-3.95)	83 (61-92)

Adjusted for age, sex, BMI, education, alcohol consumption, cigarette smoking, and study-specific variables. Abbreviations: AA, all adenocarcinoma; CI, confidence interval; EA, esophgaeal adenocarcinoma; EGJA, esophagogastric junction adenocarcinoma; OR, odds ratio; UI, uncertainty interval.

Supplementary Table 34. Associations between heartburn and/or regurgitation duration and adenocarcinomas of the esophagus in ever-cigarette smokers.

Analysis	EA		EGJA	
	OR (95%CI)	I^2 (95%UI)	OR (95%CI)	I^2 (95%UI)
**Heartburn Duration (years)**				
Never	*Referent*		*Referent*	
0.1 to <10	2.67 (1.45-4.91)	57 (0-84)	2.62 (1.62-4.26)	43 (0-79)
10 to <20	4.31 (3.11-5.97)	0 (0-75)	1.37 (0.69-2.72)	67 (15-87)
≥20	5.55 (3.32-9.26)	70 (23-88)	3.14 (1.94-5.10)	66 (10-87)
**Regurgitation Duration (years)**				
Never	*Referent*		*Referent*	
0.1 to <10	2.72 (1.37-5.42)	74 (35-89)	1.94 (0.73-5.13)	88 (76-95)
10 to <20	4.16 (2.67-6.48)	29 (0-72)	1.64 (0.83-3.22)	65 (8-87)
≥20	4.26 (2.23-8.11)	78 (48-91)	1.63 (0.96-2.79)	65 (7-87)
**Heartburn & Regurgitation Duration (years)**				
Never	*Referent*		*Referent*	
0.1 to <10	3.35 (1.36-8.26)	80 (52-91)	2.63 (0.82-8.41)	89 (78-95)
10 to <30	4.10 (2.50-6.73)	59 (0-85)	2.13 (1.14-3.99)	76 (42-90)
≥30	5.45 (2.90-10.25)	77 (45-91)	2.30 (1.34-3.95)	68 (19-88)

Adjusted for age, sex, BMI, education, alcohol consumption, cigarette smoking, and study-specific variables. Abbreviations: AA, all adenocarcinoma; CI, confidence interval; EA, esophgaeal adenocarcinoma; EGJA, esophagogastric junction adenocarcinoma; OR, odds ratio; UI, uncertainty interval.

Supplementary Table 35. Associations between heartburn and/or regurgitation frequency and adenocarcinomas of the esophagus in ever-cigarette smokers.

Analysis	EA		EGJA	
	OR (95%CI)	I^2 (95%UI)	OR (95%CI)	I^2 (95%UI)
**Heartburn Frequency**				
Never	*Referent*		*Referent*	
<monthly	0.94 (0.68-1.32)	0 (0-78)	0.66 (0.44-1.00)	36 (0-79)
Monthly to <weekly	2.62 (1.86-3.69)	0 (0-83)	1.82 (1.02-3.24)	58 (0-86)
Weekly to <daily	3.96 (2.36-6.63)	69 (21-88)	2.54 (1.71-3.79)	52 (0-82)
≥Daily	6.86 (3.96-11.89)	65 (8-87)	2.76 (1.97-3.87)	0 (0-70)
**Regurgitation Frequency**				
Never	*Referent*		*Referent*	
<monthly	0.73 (0.44-1.21)	48 (0-83)	0.55 (0.26-1.14)	80 (46-92)
Monthly to <weekly	3.14 (2.29-4.31)	0 (0-52)	1.77 (1.31-2.40)	0 (0-0)
Weekly to <daily	4.74 (2.88-7.79)	64 (4-86)	1.69 (0.87-3.27)	77 (44-90)
≥Daily	5.31 (3.74-7.54)	0 (0-71)	2.43 (1.51-3.91)	40 (0-78)
**Heartburn & Regurgitation Frequency**				
Never	*Referent*		*Referent*	
≤Weekly	1.84 (1.07-3.17)	74 (36-90)	1.34 (0.70-2.58)	86 (70-94)
>Weekly to Daily	4.99 (2.89-8.61)	70 (23-88)	2.18 (1.12-4.26)	80 (52-91)
>Daily	7.30 (4.34-12.28)	54 (0-83)	2.66 (1.86-3.80)	0 (0-75)

Adjusted for age, sex, BMI, education, alcohol consumption, cigarette smoking, and study-specific variables. Abbreviations: AA, all adenocarcinoma; CI, confidence interval; EA, esophgaeal adenocarcinoma; EGJA, esophagogastric junction adenocarcinoma; OR, odds ratio; UI, uncertainty interval.
